# Genetic diversity of *Plasmodium vivax* reticulocyte binding protein 2b in global parasite populations

**DOI:** 10.1186/s13071-022-05296-6

**Published:** 2022-06-13

**Authors:** Xuexing Zhang, Haichao Wei, Yangminghui Zhang, Yan Zhao, Lin Wang, Yubing Hu, Wang Nguitragool, Jetsumon Sattabongkot, John Adams, Liwang Cui, Yaming Cao, Qinghui Wang

**Affiliations:** 1 Department of Immunology, College of Basic Medical Science, China Medical University, Shenyang, 110122 Liaoning China; 2 Department of Blood Transfusion Medicine, General Hospital of Northern Theater Command, Shenyang, 110015 Liaoning China; 3 Department of Blood Transfusion, Yantaishan Hospital, Yantai, 264000 Shandong China; 4 Central Laboratory, The First Hospital of China Medical University, Shenyang, 110001 Liaoning China; 5 Mahidol Vivax Research Unit, Faculty of Tropical Medicine, Mahidol University, Bangkok, Thailand; 6 College of Public Health, Global Health Infectious Disease Research (GHIDR) Program, Tampa, FL USA; 7 Department of Internal Medicine, Morsani College of Medicine, University of South Florida, 3720 Spectrum Boulevard, Suite 304, Tampa, FL 33612 USA

**Keywords:** *Plasmodium v*ivax, *PvRBP2b*, Genetic diversity, Vaccine

## Abstract

**Background:**

*Plasmodium vivax* reticulocyte binding protein 2b (PvRBP2b) plays a critical role in parasite invasion of reticulocytes by binding the transferrin receptor 1. PvRBP2b is a vaccine candidate based on the negative correlation between antibody titers against PvRBP2b recombinant proteins and parasitemia and risk of *vivax* malaria. The aim of this study was to analyze the genetic diversity of the *PvRBP2b* gene in the global *P. vivax* populations.

**Methods:**

Near full-length *PvRBP2b* nucleotide sequences (190–8349 bp) were obtained from 88 *P. vivax* isolates collected from the China–Myanmar border (*n* = 44) and Thailand (*n* = 44). An additional 224 *PvRBP2b* sequences were retrieved from genome sequences from parasite populations worldwide. The genetic diversity, neutral selection, haplotype distribution and genetic differentiation of *PvRBP2b* were examined.

**Results:**

The genetic diversity of *PvRBP2b* was distributed unevenly, with peak diversity found in the reticulocyte binding region in the N-terminus. Neutrality analysis suggested that this region is subjected to balancing selection or population bottlenecks. Several amino acid variants were found in all or nearly all *P. vivax* endemic regions. However, the critical residues responsible for reticulocyte binding were highly conserved. There was substantial population differentiation according to the geographical separation. The distribution of haplotypes in the reticulocyte binding region varied among regions; even the two major haplotypes Hap_6 and Hap_8 were found in only five populations.

**Conclusions:**

Our data show considerable genetic variations of *PvRBPb* in global parasite populations. The geographic divergence may pose a challenge to PvRBP2b-based vaccine development.

**Graphical Abstract:**

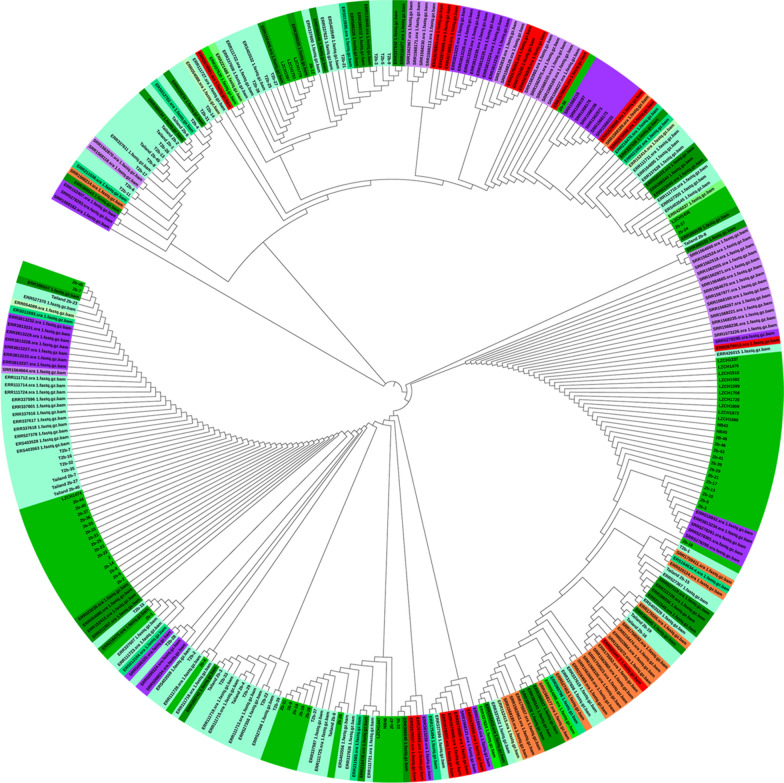

**Supplementary Information:**

The online version contains supplementary material available at 10.1186/s13071-022-05296-6.

## Background

Malaria remains a major threat to global health despite intensified control efforts in recent years. As the most widespread *Plasmodium* species, infection with *Plasmodium v*ivax caused an estimated 7.9 million malaria cases worldwide in 2018, with 53% of the *vivax* burden being in the Southeast Asia region [1]. The elimination of *P. vivax* is challenging due to its dormant liver stage, which gives rise to the relapses of malaria [2–4]. Integrated interventions, including novel tools such as vaccines, are urgently needed for malaria elimination.

The invasion of red blood cells (RBCs) by the merozoites is an essential step in the asexual erythrocytic cycle of malaria parasites [5, 6]. Since merozoites are exposed to the host immune system, vaccine candidates are usually designed to target the merozoite surface proteins, with the aim to block erythrocyte invasion [7, 8]. Compared to *P. falciparum*, much fewer vaccine candidates have been identified for *P. vivax*, partially owing to the absence of a long-term in vitro culture system for this parasite [9, 10]. *Plasmodium vivax* requires the Duffy antigen receptor for chemokines (DARC) on the surface of the RBC for invasion. However, solid evidence of *P. vivax* infection in DARC-negative individuals in Africa suggests that this *Plasmodium* species may have evolved to explore alternative pathways for invasion [11, 12]. *Plasmodium vivax* shows a restricted tropism for reticulocytes with high levels of transferrin receptor 1 (TfR1 or CD71) [13]. Recently, TfR1 has been identified as the reticulocyte-specific receptor for *P. vivax* reticulocyte binding protein 2b (PvRBP2b), a ternary complex expressed in the schizont stage [14, 15]. PvRBP2b belongs to the PvRBP family, which is composed of at least 11 members with different binding preferences for normocytes or reticulocytes [5, 16–19]. Studies on the crystal structure of the N-terminal domain of PvRBP2b have revealed a similar structural scaffold to that of *P. falciparum* reticulocyte binding protein homolog 5 (PfRh5), a well-characterized vaccine candidate for *P. falciparum* [15, 20]. In one study, monoclonal antibodies against PvRBP2b or TfR1 mutants that impede the binding of PvRBP2b to the reticulocytes successfully blocked the entry of *P. vivax* into the reticulocytes, suggesting that PvRBP2b is a promising vaccine candidate targeting blood-stage infections [15].

The N-terminal domain of PvRBP2b is responsible for reticulocyte binding [14, 15], whereas the function of the C-terminal domain is not clear. Studies with the recombinant PvRBP2b N-terminal domain have detected antibodies against PvRBP2b in plasma samples collected from *P. vivax*-infected patients, supporting the notion that PvRBP2b contains immune recognition epitopes in the N-terminal domain [15, 18, 21]. In addition, immunoglobulin G (IgG) levels against PvRBP2b have been found to be negatively correlated with parasitemia and the risk of vivax infections [18, 21, 22]. These studies have highlighted the potential of PvRBP2b as a promising target for *P. vivax* vaccine development [14, 15].

A major challenge for the efficacy of blood-stage malaria vaccines is the extensive genetic diversity of the target antigens. Therefore, understanding the genetic diversity of vaccine candidates is necessary for designing effective vaccines and predicting vaccine efficacy. In this study, we analyzed the genetic diversity, phylogenetic relationship and population differentiation of *PvRBP2b* from 312 global *P. vivax* isolates, with the aim to provide the necessary information for PvRBP2b-based vaccine development.

## Methods

### Sample collection and ethics statements

This study used 88 dried blood spots on filter papers collected from *P. vivax*-infected patients attending the Laiza and Nabang hospitals in the China–Myanmar border area in 2014 (*n* = 44) and the Tha Song Yang hospital in western Thailand in 2011–2012 (*n* = 44). Malaria was diagnosed by microscopic examination, and finger-prick blood samples were collected.

### *PvRBP2b* gene amplification and sequencing

Genomic DNA was extracted from the dried blood spots on filter papers using the QIAamp DNA Mini kit (Qiagen, Hilden, Germany). The studied parasites were part of the clinical samples genotyped by PCR–restriction fragment length polymorphism analysis of the polymorphic *Msp3α* and *Msp3β* genes in a previous study [23]. Only monoclonal infections were used for *PvRBP2b* sequencing. An 8160-bp fragment of the full-length protein-coding sequence of the *PvRBP2b* gene (8421 bp), i.e. *PvRBP2b*_*190–8349*_, corresponding to amino acids 64–2783 of the PvRBP2b protein was amplified from all the samples using KOD-Plus-Neo polymerase (Toyobo, Osaka, Japan). Given the large size of the gene, seven overlapping 1.5-kb fragments were amplified from each sample using seven pairs of primers (Additional file 1: Table S1). PCR amplification was carried out in a total reaction volume of 30 μl containing 1× KOD-Plus-Neo buffer, 200 µM dNTPs, 1 mM MgSO_4_, 250 nM primer, 0.4 units KOD Plus polymerase and 2 µl genomic DNA. The following cycling parameters were used: an initial denaturation at 94 °C for 5 min, followed by 35 cycles of denaturation at 94 °C for 15 s, annealing at a determined temperature (Additional file 1: Table S1) for 15 s, and extension at 68 °C for 90 s, with a final final extension at 68 °C for 5 min. The PCR products were separated in 1% agarose gels and then subjected to DNA sequencing using the ABI BigDye™ Terminator Reaction Ready kit (Applied Biosystems, Thermo Fisher Scientific, Waltham, MA, USA). The nucleotide sequences were deposited in the GenBank database under the accession numbers OM715042–OM715085 and OM715086-OM715129.

### Sequence assembly and retrieval

The 88 *PvRBP2b* sequences were assembled using DNASTAR Lasergene software (DNASTAR, Madison, WI, USA ). In addition, 224 *PvRBP2b* sequences from 11 global locations were obtained from previous whole-genome sequencing projects [24–29]. Fastq files were downloaded from the Sequence Read Archive (SRA) of the National Center for Biotechnology Information. We used two parameters to exclude low-quality variants: (i) quality ≤ 40 and (ii) minor allele frequency < 0.01. Only single nucleotide polymorphism (SNP) variants from monoclonal infections were included. After filtering out unqualified sequences, the global sample set of sequences included those from Brazil (*n* = 36), Colombia (*n* = 28), Cambodia (*n* = 35), China–Myanmar border (*n* = 19), Ethiopia (*n* = 18), Indonesia (*n* = 3), Laos (*n* = 2), Malaysia (*n* = 4), Papua New Guinea (PNG) (*n* = 20), Thailand (*n* = 48) and Vietnam (*n* = 11). Isolate codes and SRA accession numbers of samples used in this analysis are given in Additional file 1: Table S2.

### Analysis of genetic diversity and tests for detecting selections

A total of 312 *PvRBP2b* sequences were aligned with the reference sequence from the Salvador I (Sal I) strain (PVX_094255) using the Clustal W program in MEGA7 software. For the evaluation of *PvRBP2b* genetic diversity, the nucleotide diversity (*π*), the number of haplotypes (H) and haplotype diversity (Hd) were computed using DnaSP v5.10 software [30]. To test the departure from neutrality, Tajima’s *D* test [31], Fu and Li’s F* test [32] and Fu and Li’s D* test [32] were computed using DnaSP v5.10 software. Natural selection was determined by calculating the ratio of nonsynonymous (dN) to synonymous (dS) substitutions per nucleotide site (dN-dS), using the Nei-Gojobori method [33] with Jukes-Cantor correction for multiple substitutions. Statistical significance of the difference was estimated using the codon-based Z-test of selection implemented in MEGA 7 [33].

Finally, to determine the existence of specific codons targeted by selection in the global population [34], the FUBAR [35] and SLAC [36] methods, implemented in the Datamonkey webserver (http://www.datamonkey.org), were performed. Before analysis, putative recombination joints and parental sequences were determined using the RDP4 suite [37]. Recombination breakpoints were detected using seven methods (RDP, GENECONV, BOOTSCAN, MAXCHI, CHIMAERA, SISCAN and 3SEQ) in the RDP4 package. The probability of a putative recombination event was corrected by a Bonferroni procedure with a cutoff of *P* < 0.01. Only events supported by at least four of the seven methods were considered to be recombinants. Recombinants were then removed from the phylogenetic tree constructed by the maximum likelihood method implemented in MEGA7 using the nucleotide substitution Hasegawa-Kishino-Yano (HKY)+G+I model. As a last step, the prepared dataset was uploaded to the Datamonkey webserver for analysis.

### Population differentiation, structure and phylogenetic relationship

To investigate population subdivision, Wright’s fixation index (*F*_*ST*_) representing inter-population variance in allele frequencies was calculated using DnaSP v5.10 [30, 38, 39]. The genetic structure of all the *vivax* parasite populations was then elucidated using STRUCTURE v2.3.2 software based on the Bayesian analysis and admixture model [40, 41]. All samples were run at *K* = 2–7 (10 iterations each) with a burn-in period of 20,000 iterations followed by 1,200,000 Markov Chain Monte Carlo iterations. Then the optimal number of grouping was determined by ΔK using STRUCTURE HARVESTER v0.6.94 software [42, 43]. The partition of the clusters was presented using CLUMPP v1.1.2 [44] and the DISTRUCT 1.1 tools [45]. To determine the relationship among the parasites, phylogenetic analyses were performed using the neighbor-joining (NJ) method implemented in MEGA7 [33], and the phylogenetic tree was then optimized with the online tool ITOL [46]. A haplotype network based on the polymorphic sites in the reticulocyte binding region of *PvRBP2b* was constructed using the PHYLOVIZ 2.0 software with the NJ method [47].

### Prediction of linear B-cell epitopes

The potential linear B-cell epitopes were predicted using the BCPreds prediction tool (http://ailab-projects1.ist.psu.edu:8080/bcpred/predict.html) [48]. Antigenicity was predicted using the VaxiJen v2.0 online tool (http://www.ddgpharmfac.net/vaxijen/VaxiJen/VaxiJen.html) [49]. BCPreds predicts a peptide length of 12 consecutive amino acids with a threshold of 0.8, whereas VaxiJen sets a threshold at 0.5. The overlapped regions of predicted linear B-cell epitopes by both methods were selected. The predicted three-dimensional (3D) structures of the reticulocyte binding region of PvRBP2b were constructed with the PHYRE2 algorithm [50] and further visualized and modeled using the molecular modeling tool PyMOL V2.3 [51]. The PvRBP2b amino acid sequence in the reference Sal I strain was used for prediction.

## Results

### Mutations revealed from global *PvRBP2b* sequences

**Fig. 1:**
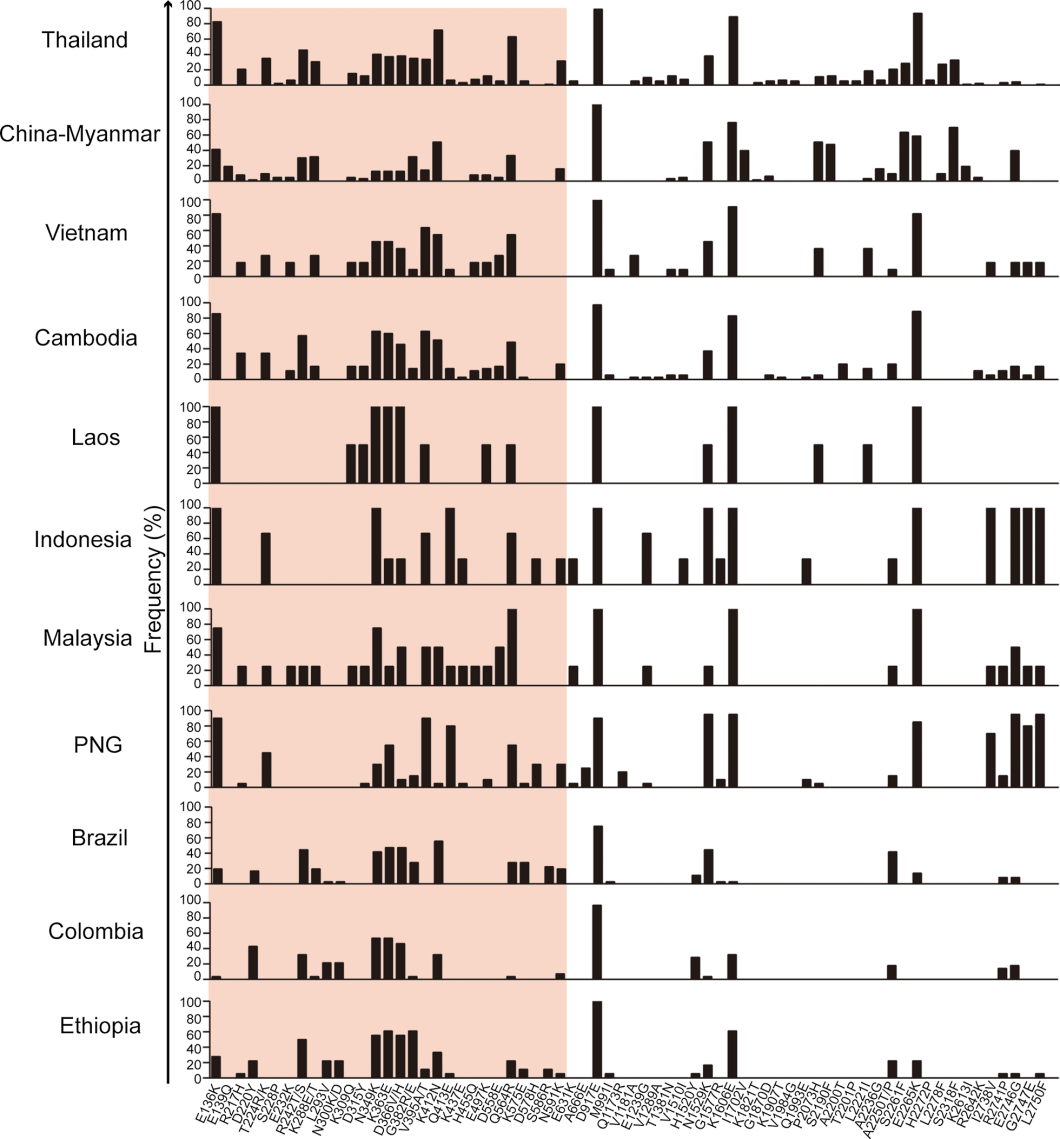
Prevalence of amino acid substitutions in PvRBP2b among worldwide *P. vivax* populations. Positions and frequencies of amino acid changes in PvRBP2b among different populations are shown. The reticulocyte binding region of PvRBP2b (168–633 amino acids) is shaded. Abbreviations: PvRBP2b, *Plasmodium vivax* reticulocyte binding protein 2b

Sequencing of the 8160-bp *PvRBP2b* fragment (190–8349 bp) was successful for the 88 *P. vivax* field isolates collected from the China–Myanmar and Thailand–Myanmar border areas. To gain a global perspective, we retrieved 224 *PvRBP2b* sequences from the whole-genome sequences of *P. vivax* isolates collected in multiple *P. vivax* endemic areas across the world. A preliminary analysis showed that the nucleotide diversity of sequences obtained by Sanger sequencing from the China-Myanmar border (0.00167) and Thailand (0.00217) was comparable to that obtained from the whole-genome sequencing projects (0.00116 and 0.00148, respectively). Therefore, the combined data were used for analysis. Alignment of all 312 *PvRBP2b* sequences with the Sal I reference identified 116 SNPs, including 103 nonsynonymous and 20 synonymous mutations. Of note, the 103 nonsynonymous mutations resulted in 96 amino acid changes. Of all nonsynonymous mutations, 75 SNPs showed allele frequencies of > 1% (Additional file 1: Table S3). The distributions of the 75 mutations in different areas are shown in Fig. 1 and Additional file 1: Table S3. The E136K, N349K, K363E, D366V/H, V395A/T, K412N, Q564R, D917E, N1529K, K1606E, E2265K and E2746G amino acid mutations were found in all or nearly all endemic sites, reflecting the high prevalence of *PvRBP2b* polymorphisms in the world. Among these, D917E approached fixation (95.5%).

**Fig. 2:**
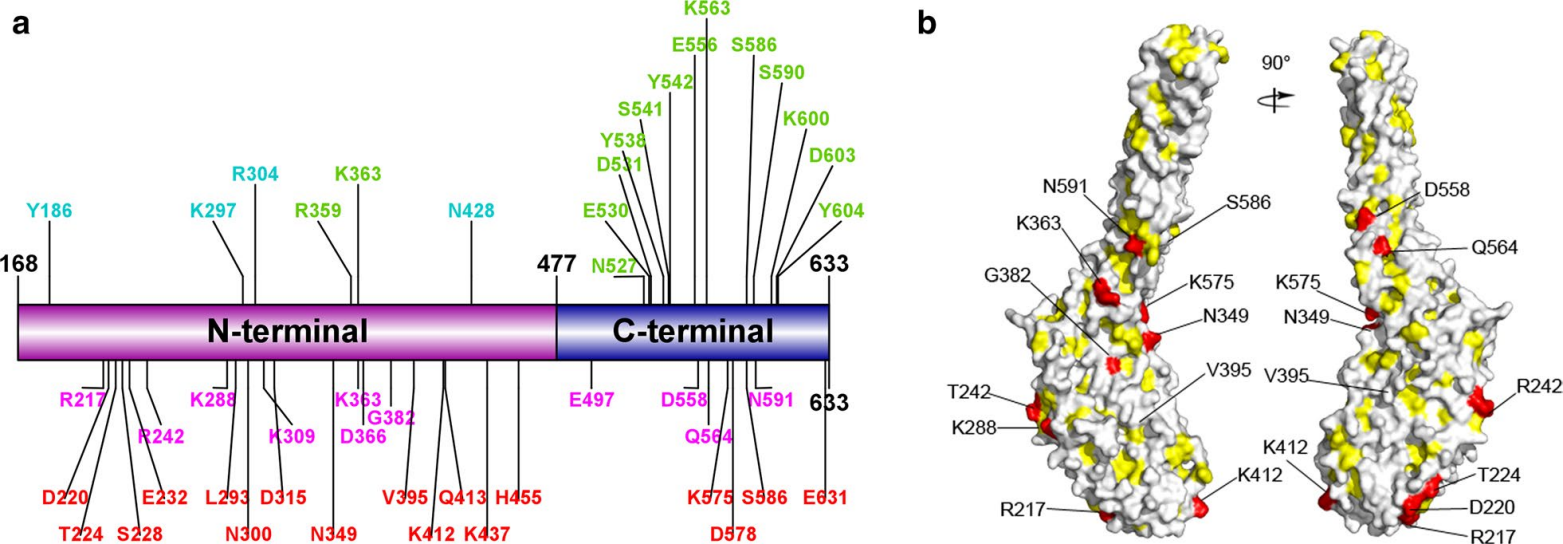
Three-dimensional structure of the PvRBP2b reticulocyte binding region. **a** A modified schematic diagram from [14] shows the important residues in the reticulocyte binding region composed of the N-terminal and C-terminal domains. The polymorphic residues previously reported are labeled in magenta, whereas the polymorphic residues newly identified in the present study are labeled in red. Residues interacting with TfR1 and Tf are shown in green and cyan, respectively. **b** Two orthogonal views of the three-dimensional model structure of the PvRBP2b reticulocyte binding region shows the 14 residues positively selected by both codon-based tests in the Datamonkey webserver and S586 positively selected by FUBAR. The hydrophobic binding region is shown in yellow. Abbreviations: Tf, transferrin; TfR1 transferrin receptor 1

The reticulocyte binding region on the N-terminus of PvRBP2b is critical for receptor binding and RBC invasion. It corresponds to the amino acids 168–633 since its flanking regions failed to be visualized in the PvRBP2b structure [14]. As shown in Additional file 1: Table S3, from a total of 75 nonsynonymous nucleotide substitutions with allele frequencies > 1%, almost 50% (35/75) were in the reticulocyte binding region, generating 28 amino acid mutations. In addition to the 11 nonsynonymous mutations (R217H, R242T/S, K288P, K309Q, K363E, D366V/H, G382R/E, E497K, D558E, Q564R and N591K) previously reported for the reticulocyte binding region [14], 17 additional nonsynonymous mutations (D220Y, T224R/K, S228P, E232K, L293V, N300K/D, D315Y, N349K, V395A/T, K412N, Q413E, K437E, H455Q, K575E, D578H, S586R and E631K) were reported in the present study (Fig. 2a). Among these, K363E and S586R are the residues known to interact with the receptor TfR1 [14]. K363E was prevalent in all endemic areas, with an allele frequency of 40.4%, whereas S586R was found only in Brazil (22.2%), Ethiopia (11.1%) and Thailand (1.1%) (Additional file 1: Table S3).

### Genetic diversity of *PvRBP2b*

**Fig. 3:**
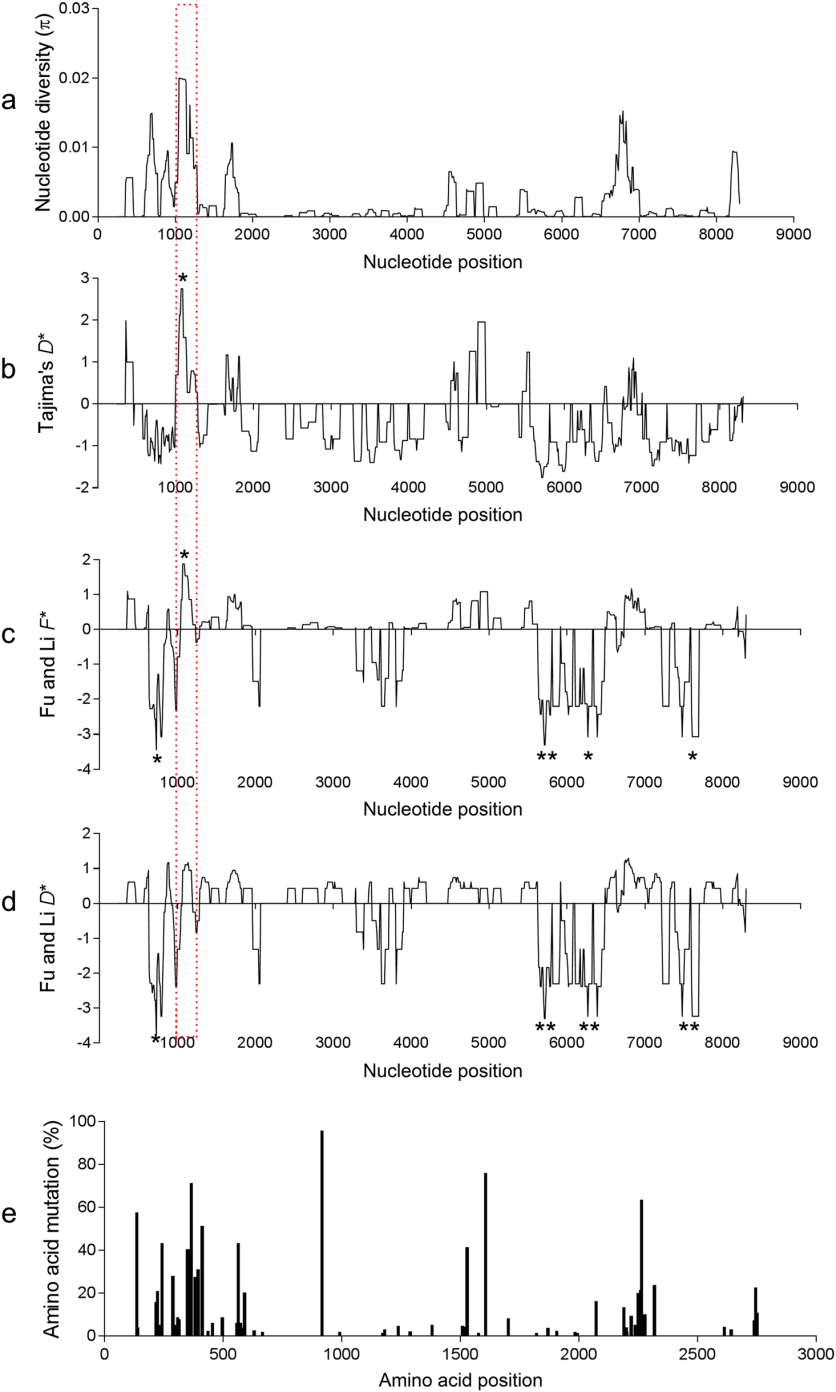
Nucleotide diversity, neutrality tests and amino acid polymorphisms. Sliding window plots of nucleotide diversity (*π*) (**a**), Tajima’s D* (**b**), Fu and Li’s F* (**c**) and Fu and Li’s D* (**d**) for *PvRBP2b* sequences are shown with a window size of 90 bp and a step size of 3 bp. Amino acid polymorphisms (**e**) are also visualized to the corresponding locations. The asterisk (*) depicts sites of statistical significance under balancing or directional selections (*P* < 0.05)

The population genetic indices were analyzed to assess the nucleotide diversity (*π*) of *PvRBP2b* (Table 1). The overall nucleotide diversity from 312 sequences was 0.00196, with the highest found in the population from Malaysia (0.00203), followed by Thailand (0.00198) and Vietnam (0.00193). The overall haplotype diversity (Hd) was high (0.997) (Table 1). The sliding window plots of nucleotide diversity revealed an uneven distribution, with the peaks located at nucleotides 1015–1134 within the reticulocyte binding region (Fig. 3a). Similarly, 46.7% of amino acid substitutions were clustered in the reticulocyte binding region (Fig. 3e). This result reflects the relatively high polymorphism in the reticulocyte binding region of *PvRBP2b* in the worldwide *P. vivax* populations.

**Table 1:** Genetic diversity of near-full length *Plasmodium vivax* reticulocyte binding protein 2b gene in global populations

Populations	No. of isolates	No. of polymorphic sites	*π* ± SD	H	Hd ± SD
Brazil	36	30	0.00121 ± 0.00089	18	0.930 ± 0.023
Colombia	28	29	0.00112 ± 0.00091	18	0.950 ± 0.025
Cambodia	35	55	0.00172 ± 0.00167	30	0.987 ± 0.012
China–Myanmar	63	81	0.00178 ± 0.00213	45	0.973 ± 0.012
Ethiopia	18	32	0.00120 ± 0.00114	16	0.980 ± 0.028
Laos	2	9	0.00110 ± 0.00110	2	1.000 ± 0.500
Malaysia	4	31	0.00203 ± 0.00208	4	1.000 ± 0.177
Papua New Guinea	20	39	0.00114 ± 0.00135	20	1.000 ± 0.016
Thailand	92	97	0.00198 ± 0.00244	85	0.998 ± 0.002
Vietnam	11	43	0.00193 ± 0.00184	11	1.000 ± 0.039
Indonesia	3	16	0.00131 ± 0.00131	3	1.000 ± 0.272
Total	312	147	0.00196 ± 0.00299	248	0.997 ± 0.0008

*H* Number of haplotypes, *Hd* haplotype diversity, *SD* standard deviation,*π* nucleotide diversity

### Evidence of potential selections

Neutrality tests were conducted to evaluate whether the *PvRBP2b* gene followed the neutral equilibrium model of molecular evolution. Although the overall *PvRBP2b* sequence did not significantly deviate from neutrality, a sliding window analysis identified significant positive values for the 1015- to 1034-bp region by Tajima’s D* and Fu and Li’s F* tests, which paralleled the peak* π* value, reflecting balancing selection within the reticulocyte binding region or population bottlenecks in the global populations (Fig. 3b–d). In addition, some significant negative values for several C-terminal domains suggested population expansion or excess of singletons (Fig. 3b–d). The dN-dS statistic was positive for the reticulocyte-binding region among the global populations (Table 2), suggesting that polymorphisms found for this region of *PvRBP2b* were maintained by diversifying selection.

**Table 2:** Tests for selection in the *PvRBP2b* gene from global samples

Gene fragment encoding *PvRBP2b*	*N*	Codon-based Z-test	
		dN-dS	*P* value
Near full-length sequence	312	0.995	0.322
Reticulocyte binding region	312	2.801**	0.006

*N* Number of isolates

^**^Significant at *P* < 0.01

The likelihood-based algorithms were used to determine specific codons targeted by selection. Only one recombinant identified by the RDP, GENECONV, MAXCHI, CHIMAERA, SISCAN and 3SEQ methods implemented in the RDP suite was removed from the phylogenetic and selection analyses (Additional file 1: Table S4). Thirty-two positively selected and 11 negatively selected codons were identified by both the SLAC and FUBAR methods (Table 3). Fourteen of the positively selected mutations are located in the reticulocyte-binding region, presumptively associated with parasite invasion and/or immune recognition (Table 3). K363 and S586 have been shown to be the reticulocyte-binding sites [14]. In contrast to the positive selection at K363 confirmed by all three methods, S586 was supposed to be positively selected only by the FUBAR method (Table 3). Meanwhile, sites under purifying selection were scattered along the PvRBP2b (Table 3).

**Table 3:** Codon-based tests for selection in *PvRBP2b* gene

Selection sites	SLAC method	FUBAR method	By both algorithms
Positive/diversifying selection sites	136,217,220,224,242,288,349,363,382,395,412,558,564,575,591,917,1239,1381,1510,1529,1606,2073,2190,2200,2221,2250,2261,2265,2278,2318,2741,2746	136,217,220,224,228,232,242,288,293,300,309,315,349,363,382,395,412,413,437,455,497,558,564,575,578,586,591,631,666,917,991,1168,1173,1181,1239,1289,1381,1510,1520,1529,1577,1606,1821,1870,1907,1984,2073,2190,2200,2201,2221,2236,2250,2261,2265,2272,2278,2318,2370,2517,2605,2613,2642,2738,2741,2746,2747,2750	136,217,220,224,242,288,349,363,382,395,412,558,564,575,591,917,1239,1381,1510,1529,1606,2073,2190,2200,2221,2250,2261,2265,2278,2318,2741,2746
Negative/purifying selection sites	366,885,1644,1834,1886,2149,2238,2247,2255,2292,2391,2465	885,1113,1644,1834,1886,1987,2149,2225,2238,2247,2255,2292,2329,2391,2465	885,1644,1834,1886,2149,2238,2247,2255,2292,2391,2465

### Mutations within the 3D structure and predicted B-epitopes in the reticulocyte binding region

The 3D model structure with positively selected amino acid mutations mapped in the reticulocyte binding region of PvRBP2b showed that most mutated residues were on the surface of α helices, with the exceptions of V395, which was hidden inside the protein, and K412, which was located in the flexible loop structure (Fig. 2b). Most mutations were close to the hydrophobic binding region. Three peptides (270–281, 519–530, and 566–577 amino acids) in the reticulocyte binding region of PvRBP2b were predicted to be linear B-epitopes with the prediction scores of 0.839, 0.987, and 0.959, respectively, by the BCPreds and VaxiJen software (Table 4). However, only one polymorphic residue, K575, was presented in the predicted B-epitope of 566–577 amino acids. Seven additional polymorphic residues were found in the predicted linear B-epitopes in other regions of PvRBP2b (Table 4).

**Table 4:** Predicted *PvRBP2b* B-cell epitopes in the reticulocyte binding region

Start position	Stop position	Sequence	Score
60	71	DDGKINDGGDEK	0.957
73	84	HSPDSSFSGDSE	0.999
107	118	SSNTNKSLNDSN	0.939
139	150	*E*KKTTKSEPAPK	0.984
156	167	PSPKEPSPESTQ	0.993
**270**	**281**	**KLRQYEEKKEAF**	0.839
**519**	**530**	**NEFKKDYDNNVE**	0.987
**566**	**577**	**NIPANSNAQ*K*KV**	0.959
902	913	ETYTEKKDEETK	0.913
943	954	SNTINEVENENK	0.923
1048	1059	KSRENIKGNNGT	0.969
1108	1119	DRDIKEKGKDIE	0.929
1237	1248	EK*E*ETTSNEVDN	0.881
1263	1274	QKINEENTKAKG	0.986
1317	1328	DKKITEIVQHAI	0.994
1330	1341	KKGEAERSKKTN	0.896
1391	1402	SKSTNEAENFEK	0.863
1523	1534	EVNKTE*N*EAEKE	0.999
1569	1580	TKIMGQIE*G*EHS	0.849
1813	1824	REEAQKEE*K*NIE	1
1888	1899	SGSSAKLQQAED	0.809
2165	2176	EKGKKCEMTKYK	0.816
2276	2287	DT*L*ENEAKMLKE	0.971
2334	2345	DQKKKLQEAKNK	0.993
2401	2412	KKGKTYEENVTH	0.839
2429	2440	EKDKLKNTNIEM	0.899
2537	2548	EKINTYIRQKIR	0.926
2642	2653	*R*EIKELEEKVYS	0.948
2702	2713	SDEGNNNDMSTT	0.997
2718	2729	EEKQTGEEESQH	1
2767	2778	DEEKKDPESVGE	0.993

The reticulocyte binding region sequence is given in bold font; polymorphic residues are shown in italics/underlining

### Haplotype network analysis

**Fig. 4:**
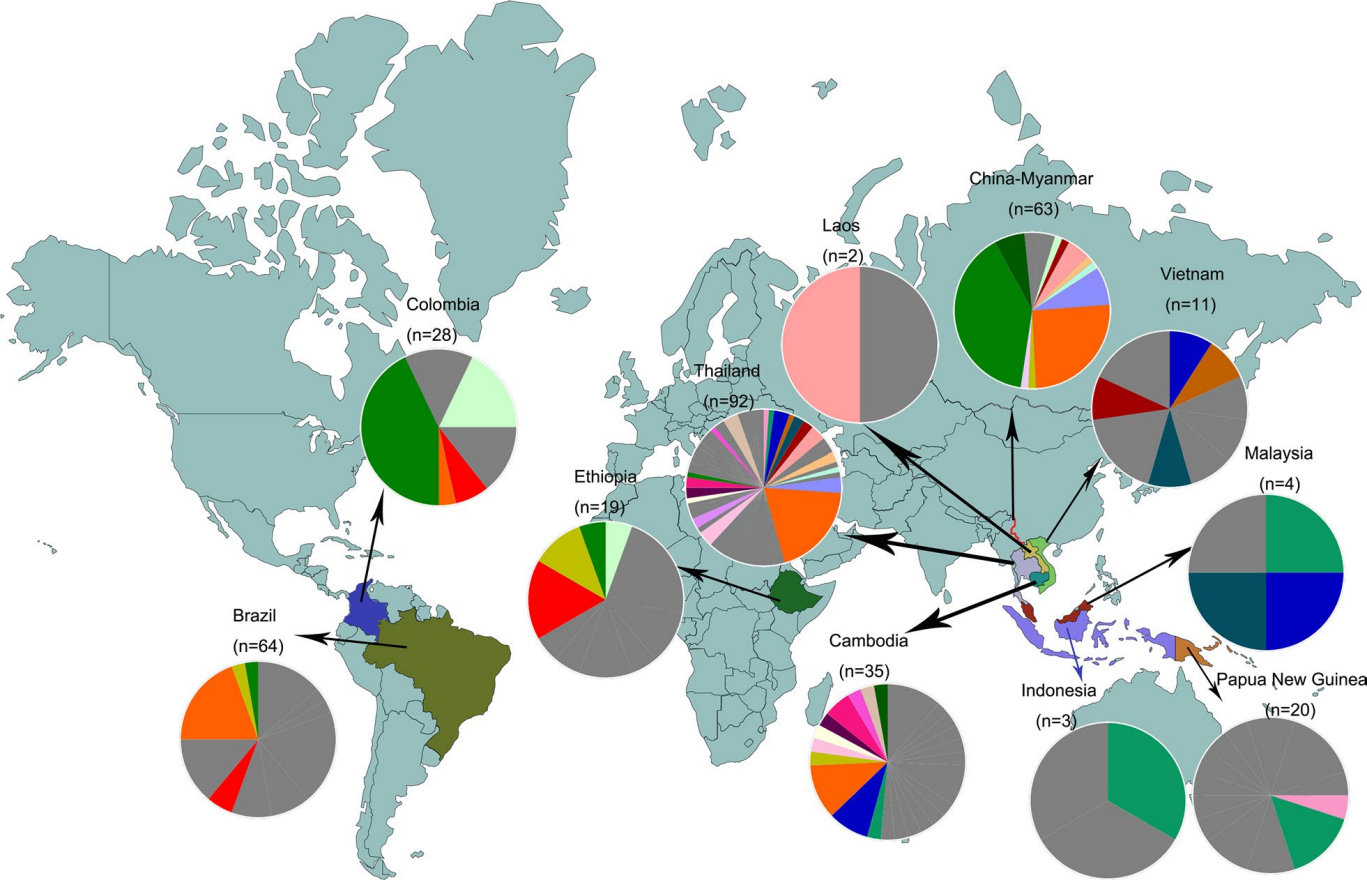
Map showing the distribution of *PvRBP2b* haplotypes. The frequencies of 114 haplotypes based on the 17 nonsynonymous amino acid mutations (> 5%) in the PvRBP2b reticulocyte binding region are depicted as pie charts and mapped to their geographical origins. The red line represents the region along the Myanmar–China border. Shared haplotypes are shown in color, and unique haplotypes are shown in gray

**Fig. 5:**
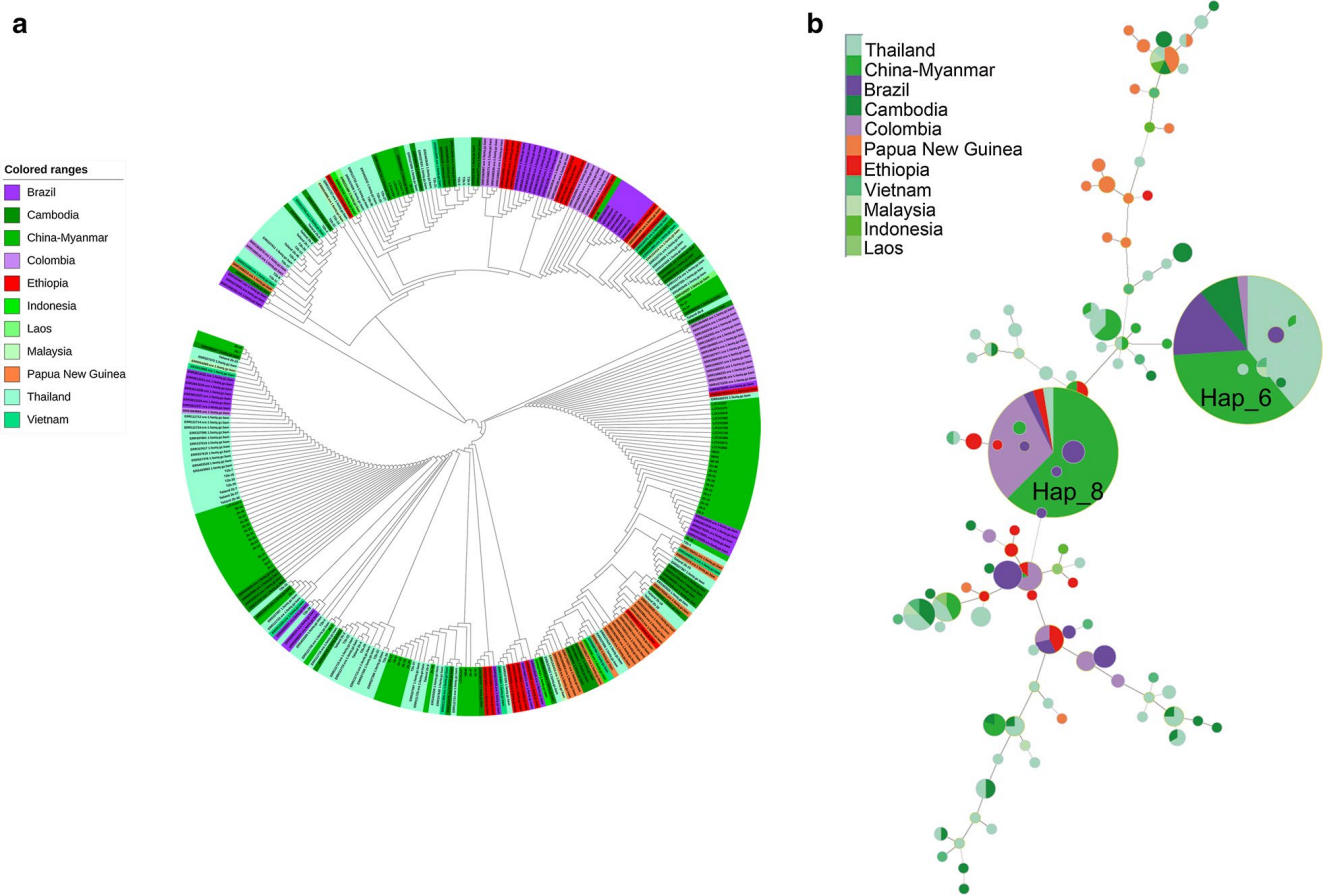
Phylogenetic relationship of the parasite isolates based on the *PvRBP2b* reticulocyte binding region. **a** An unrooted phylogenetic tree. The tree was constructed using the neighbor-joining method with bootstrap supports from 1000 replicates. The origins of the parasite isolates are represented by different colors. **b** A haplotype network. The size of the pies reflects the frequency of the specific haplotype. The lengths of the lines connecting the pies measured from their centers are in proportion to the number of base pair substitutions separating the haplotypes. Endemic regions are represented by same colors used in the phylogenetic tree

The 17 SNPs with minor allele frequencies of > 5% in the *PvRBP2b* reticulocyte binding region were chosen for haplotype analysis, which yielded a total of 114 haplotypes among the global dataset of 312 sequences (Additional file 1: Table S5; Figs. 4, 5). Of the 114 haplotypes, 58 (50.9%) were region-specific, with a frequency of between 0.32 and 1.60%. There were many rare haplotypes; 92.1% (105/114) of the haplotypes were shared by no more than five parasite isolates, among which 70/114 were represented by single parasite isolates. Even the two major haplotypes, i.e. Hap_6 and Hap_8, with a frequency of 14.74 and 12.82%, respectively, were only found in five regional populations. Hap_8, the same as the Sal I haplotype, was most abundant in the China–Myanmar border (39.7%) and Colombia (42.9%). Hap_6, the predominant haplotype identified in Thailand, was also shared among the parasite populations from the China–Myanmar border, Cambodia, Brazil and Colombia. The haplotypes in Asia were distributed in the parasite populations included in this study, and Thailand harbored the highest haplotype diversity (52/114) (Additional file 1: Table S5; Figs. 4, 5). In contrast, the distribution pattern shown in the haplotype network was different from that of parasite populations from South America (Brazil and Colombia), Africa (Ethiopia) and Oceania (Papua New Guinea [PNG]) (Fig. 5). Of the 20 parasite isolates in PNG, 16 were from a unique haplotype restricted to this specific region (Additional file 1: Table S5; Fig. 4).

### Population structure and differentiation

**Fig. 6:**
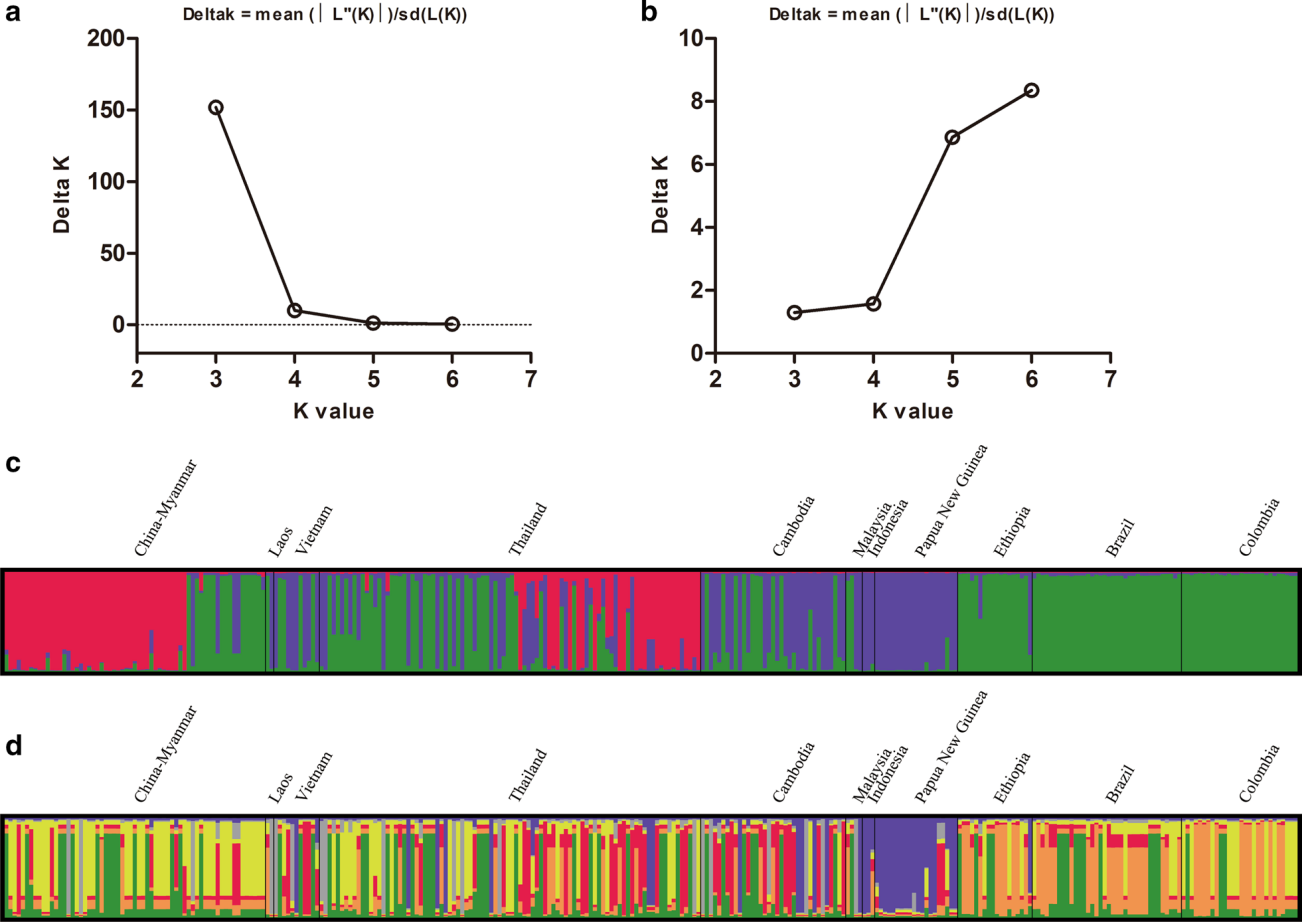
Structure analysis of *PvRBP2b* sequences from the global *P. vivax* populations. Plots represent the relationship between Delta* K* and* K* values for the near full-length sequences of PvRBP2b (**a**) and for the reticulocyte binding region (**b**), as well as the genetic structures of the near full-length sequences of *PvRBP2b* at *K* = 3 (**c**) and of the reticulocyte binding region at *K* = 6 (**d**) in parasite populations from different endemic regions

Analysis of the *PvRBP2b* sequences showed that the global parasites were optimally grouped into three clusters (*K* = 3) (Fig. 6a, c). These clusters were unevenly distributed among different geographical regions. The parasites from PNG and Indonesia were mainly represented by the purple cluster, whereas the parasites from Ethiopia, Brazil and Colombia occupied the green cluster. The red cluster represents parasites predominantly from the China–Myanmar border and Thailand. Interestingly, the remaining parasites from the China–Myanmar border and Thailand and parasites from other countries of the Greater Mekong Subregion (GMS) (Cambodia, Laos and Vietnam) showed genetic mixing of the green and purple clusters. When structure analysis was conducted using the *PvRBP2b* reticulocyte binding region, six clusters were identified with overlapping, worldwide distribution (Fig. 6b, d). It is noteworthy that only the parasites from the PNG and Indonesia formed genetically distinct clusters from other geographical regions.

**Fig. 7:**
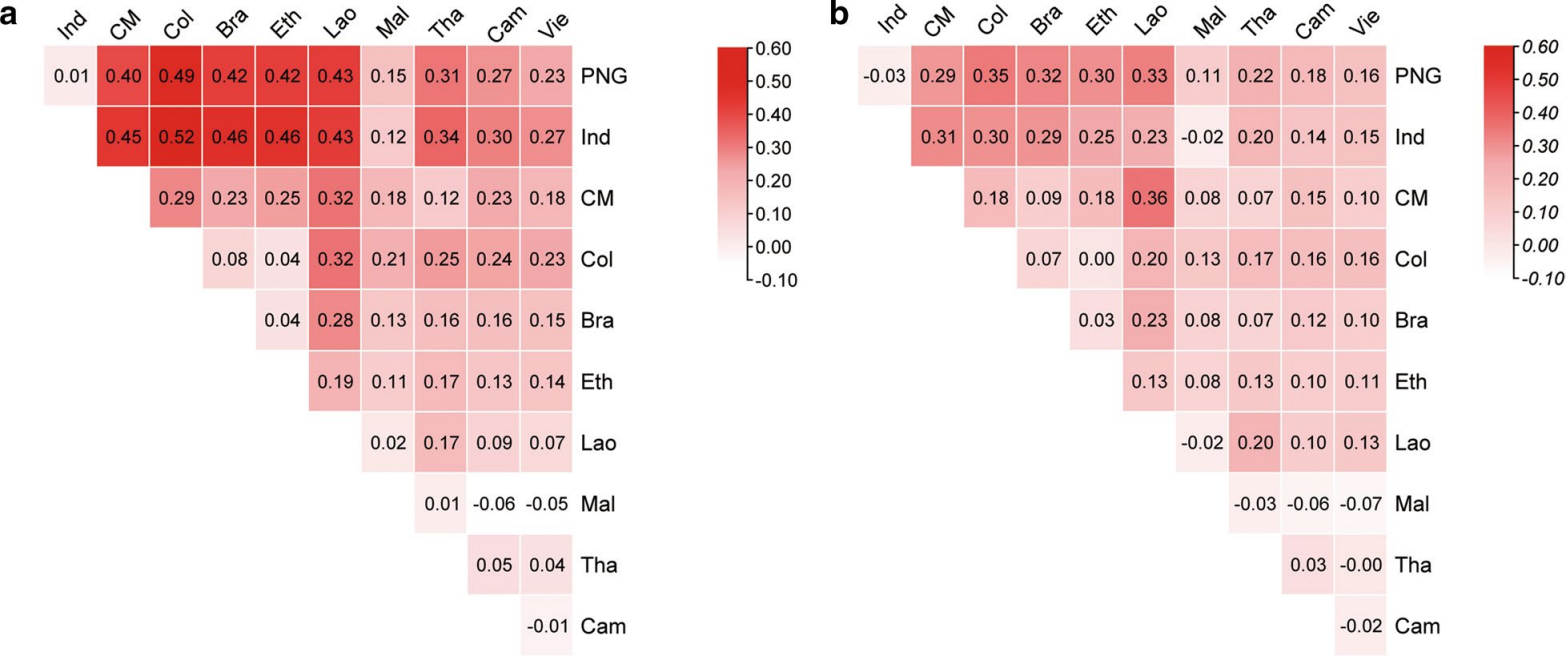
*F*_ST_ analysis of global *P. vivax* populations. Heatmaps show pairwise comparison for the near full-length sequence (**a**) and the reticulocyte binding region (**b**) in *PvRBP2b* among worldwide *P. vivax* populations. The numbers in the cells are the *F*_*ST*_ values between two endemic areas. Abbreviations: CM, China–Myanmar border; *F*_ST_, Wright’s fixation index

The genetic differentiation between two parasite populations was also evaluated via Wright’s fixation index (*F*_ST_) using the entire *PvRBP2b* sequence and reticulocyte binding region, respectively. The heatmap of the *F*_ST_ values from both analyses revealed population differentiation patterns consistent with the structure analysis (Fig. 7). Consistent with the principle of isolation by distance, parasite populations were genetically similar within each continent (e.g. Brazil vs Colombia, PNG vs. Indonesia, countries within the GMS). In contrast, considerable differentiation was detected between populations from different continents. Notably, the PNG and Indonesia *P. vivax* populations had high levels of genetic differentiation from the rest of the global parasite populations. In comparison, parasite populations from the GMS were moderately differentiated from the South American parasite populations. Interestingly, parasites from Africa (represented by Ethiopia) showed little genetic differentiation from the GMS and South American parasites.

## Discussion

In the present study, we evaluated the genetic diversity of *PvRBP2b* as a *P. vivax* malaria blood-stage vaccine candidate. The overall nucleotide diversity of *PvRBP2b* from the global samples was modest (0.00196), much lower than that of the highly polymorphic surface antigens, such as *PvAMA1* (0.0093, *n* = 372) and *PvMSP1* (0.0023–0.0495 for the conserved blocks and 0.1193–0.2055 for the variable blocks, *n* = 40) [52, 53]. The genetic polymorphisms of *PvRBP2b* were geographically heterogeneous, with higher diversity found in GMS countries than in South America (Brazil and Colombia), Africa (Ethiopia) and Oceania (PNG). A similar genetic diversity distribution pattern was also observed in *PvAMA1*, and it seemed to correlate with the larger effective population size in Southeast Asia [52]. The authors of a previous study speculated that *P. vivax* in Southeast Asia was possibly the source population [54]. The high transmission intensity and the frequent migrations of infected people among Southeast Asian countries might result in the large effective population size in this region. Recent continuous malaria control and elimination strategies successfully reduced the parasite incidence, but these seemed to have little short-term effect on the *P. vivax* population size [55, 56]. The relatively high levels of asymptomatic* vivax* infections in endemic populations possibly contributed to the maintenance of the effective population size [57, 58].

PvRBP2b-mediated reticulocyte invasion depends on the *PvRBP2b*–TfR1–Tf ternary complex [14]. Consistent with previous reports [15], we found that the reticulocyte binding region was under balancing selection; it accumulated most of the amino acid substitutions and had the highest diversity. Interestingly, most of the residues on the reticulocyte binding region were conserved. At least three residues (Y542, K600 and Y604) on PvRBP2b were critical to the reticulocyte binding and complex formation: mutations at these sites reduced binding affinity by around 80% [14]. These amino acids were absolutely conserved among all 312 samples, reflecting their essential function in reticulocyte binding. Most of the identified residues making contact with TfR1/Tf based on structure analysis [14] were conserved, with the exception of S586R and K363E, which had low (3.5%) and moderate (40.4%) allele frequencies, respectively. S586R had a limited distribution in Brazil, Ethiopia and Thailand, whereas K363E occurred in all *P. vivax* endemic areas*.* Additional nonsynonymous SNPs of low-modest frequencies (1.6–51%) were detected surrounding the interaction sites. Several residues within the reticulocyte binding region were under positive selection, raising the possibility that they resulted from the host immune selection [59]. Moreover, most of the positively selected residues are located on the α helices, some being close to the hydrophobic binding region. The K412N mutation had a frequency of 51.3% and resided in the flexible loop structure. Only one mutation, K575E, was mapped to the predicted linear B-cell epitope. However, it is still unclear whether these specific polymorphic residues would change protein structure by altering protein polarity and hydrophilicity, therefore adapting to the different TfR1 mutants (e.g. L212V, N348A, and S412G) [14, 60, 61]. More functional investigations are required to answer these questions.

One of the obstacles hindering the development of a successful malaria vaccine is the extensive genetic diversity of blood-stage antigens in *P. vivax* [62]. Naturally acquired antibodies against the reticulocyte binding region of PvRBP2b showed a strong association with reduced parasitemia [18, 21, 22], highlighting the vaccine potential of PvRBP2b. Although the overall genetic diversity of *PvRBP2b* was not as high as that of leading blood-stage vaccine candidates, *PvAMA1* and *PvMSP1*, the testing of which has advanced to clinical trials [63], the reticulocyte binding region of PvRBP2b deserves further attention. This region carried almost 50% of the mutations in the entire PvRBP2b, and analysis of the global parasite samples identified 114 haplotypes. Furthermore, most haplotypes were region-specific and represented by a single parasite isolate, and very few haplotypes were shared worldwide. Even the predominant haplotypes Hap_6 (14.74%) and Hap_8 (12.82%) were distributed in only five regions. Of note, haplotype patterns in PNG were distinct from those of other worldwide populations. This enormous haplotype diversity may present a challenge to developing a PvRBP2b-based vaccine. Since an effective vaccine should include most of the common alleles relevant to the induction of immune responses to ensure sufficient coverage of the genetic diversity [64, 65], it is essential to determine whether the major PvRBP2b alleles confer strain-transcending immunity.

This study provides several lines of evidence confirming the geographical separation of global *P. vivax* populations. Pairwise *F*_*ST*_ comparison identified the considerably high levels of differentiation between the Oceania (PNG and Indonesia) parasite populations and other *P. vivax* endemic regions, whereas parasite populations in the GMS were much less differentiated. Interestingly, populations from South America appeared to be closely related to those from Africa (Ethiopia). Population structure analysis further reinforced this finding on population relatedness. The population differentiation pattern identified in the present study is in agreement with that reported in other population studies using individual genes [66], microsatellites [54, 67] or whole-genome sequences [24, 25, 68]. The distinct parasite genetic structure in PNG may reflect the limited gene flow between PNG and the rest of the world, while the unique RBC polymorphisms in the human populations may have also contributed to this difference [52, 67, 69, 70]. In the GMS, however, the high transmission intensity in some border areas and frequent host migrations among the different countries are likely responsible for the panmixia of parasite populations. It should be noted that this study was biased, with most of the samples originating from Southeast Asia and, consequently, the conclusions may not represent global parasite populations. Population genetics can help assess the effects of malaria control strategies, track the source of imported infections, and inform the vaccine design.

## Conclusion

In conclusion, this study revealed a remarkably high level of genetic polymorphisms in *PvRBP2b* among the *P. vivax* populations in different endemic areas, with mutations clustered in the reticulocyte binding region. The genetic differentiation of parasite populations among different continents was notable, suggesting a potential need to cover major protein variants in case of strain-transcending immunity. Future studies addressing the functions of antibodies against different PvRBP2b variants are warranted.

## Supplementary Information

**Additional file 1: Table S1.** Primers used for *PvRBP2b* gene amplification and sequencing. **Table S2.** Accession number, country of origin and referenced study for the 224* P. vivax* isolates analyzed in this study. **Table S3.** Genetic mutations of near-full length *PvRBP2b* in different areas. **Table S4.** Recombination events detected in the near full-length PvRBP2b gene using the RDP4 package. **Table S5.** Sequences and distribution of *PvRBP2b* reticulocyte binding region haplotypes.

## Data Availability

The data supporting the conclusion of this article are included within the article. Representative sequences are submitted to the GenBank database under the Accession Numbers: OM715042-OM715085 and OM715086-OM715129.

## Abbreviations

DARC: Duffy antigen receptor for chemokines
*F*_ST_: Wright’s fixation index
GMS: Greater Mekong subregion
H: Number of haplotypes
Hd: Haplotype diversity
PfRh5: *Plasmodium falciparum* reticulocyte binding protein homolog 5
PNG: Papua New Guinea
PvAMA1: *Plasmodium vivax* apical membrane antigen
PvMSP: *Plasmodium vivax* merozoite surface protein 1
PvRBP2b: *Plasmodium vivax* reticulocyte binding protein 2b
RBCs: Red blood cells
SNPs: Single nucleotide polymorphisms
SRA: Sequence read archive
TfR1: Transferrin receptor 1
*π*: Nucleotide diversity
