# Supplementary material for: Genetic diversity of Plasmodium vivax reticulocyte binding protein 2b in global parasite populations
Source: Parasit Vectors. 2022 Jun 13;15:205. doi: 10.1186/s13071-022-05296-6 (PMC9191549; doi:10.1186/s13071-022-05296-6)
Supplement: Supplementary file 1 — Additional file 1: Table S1. Primers used for PvRBP2b gene amplification and sequencing. Table S2. Accession number, country of origin and referenced study for the 224 P. vivax isolates analyzed in this study. Table S3. Genetic mutations of near-full length PvRBP2b in different areas. Table S4. Recombination events detected in the near full-length PvRBP2b gene using the RDP4 package. Table S5. Sequences and distribution of PvRBP2b reticulocyte binding region haplotypes. [file 13071_2022_5296_MOESM1_ESM.docx]

Table S1. Primers used for *PvRBP2b* gene amplification and sequencing

| Primers | 5' to 3' DNA sequence | Annealing temperature (℃) |
| --- | --- | --- |
| 1-F | TGATGATGGCAAAATTAACGATGGAGGT | 60 |
| 1-R | GTTACAAATGCCGATTGCTCTGGATGCT |  |
| 2-F | CGCATCATATCACACAGAAATCAAAC | 57 |
| 2-R | CGCTCGTGAAATGTATGCATTATTTC |  |
| 3-F | CCAACAACATGTGGAAGAATTAATCAAC | 59 |
| 3-R | CCTGTATACGTTGCTCAACTTTAAGCTC |  |
| 4-F | CGCAAGCGAAATTGAGTGGGAAGCTAC | 62 |
| 4-R | CCCCCTCAATTTGGCCCATTATTTTGG |  |
| 5-F | GCCGACTGTGTGGAACACTCAAAAG | 62 |
| 5-R | GTAGGCGGCTTGCACATTTTCGTGGAC |  |
| 6-F | GAGCCTAAACGATGAAAGTGGCTCG | 64 |
| 6-R | CGCTTTAATGGACACTAGGGTGAAGG |  |
| 7-F | GAACTGCTTGGCGAAGTAGTAAAGGATC | 58 |
| 7-R | GAATAATCGCTATTGTCGAAGCAAACGT |  |

Table S2. Accession number, Country of origin and referenced study for the 224 P. vivax isolates analyzed in this study.

| **No.** | **SRR/ERS sample accession** | **SRP/ERR study accession** | **References** | **Continent** | **Country** |
| --- | --- | --- | --- | --- | --- |
| 1 | SRR5098034 |  | de Oliveira et al. 2017 | South America | Brazil |
| 2 | SRR5099324 |  | de Oliveira et al. 2017 | South America | Brazil |
| 3 | SRR5099325 |  | de Oliveira et al. 2017 | South America | Brazil |
| 4 | SRR5278293 |  | de Oliveira et al. 2017 | South America | Brazil |
| 5 | SRR5278295 |  | de Oliveira et al. 2017 | South America | Brazil |
| 6 | SRR5278298 |  | de Oliveira et al. 2017 | South America | Brazil |
| 7 | SRR5278291 |  | de Oliveira et al. 2017 | South America | Brazil |
| 8 | SRR5278301 |  | de Oliveira et al. 2017 | South America | Brazil |
| 9 |  | ERR019040 | de Oliveira et al. 2017 | South America | Brazil |
| 10 | SRR1568192 |  | de Oliveira et al. 2017 | South America | Brazil |
| 11 | SRR1564664 |  | de Oliveira et al. 2017 | South America | Colombia |
| 12 | SRR1568235 |  | de Oliveira et al. 2017 | South America | Colombia |
| 13 | SRR1568128 |  | de Oliveira et al. 2017 | South America | Colombia |
| 14 | SRR1573226 |  | de Oliveira et al. 2017 | South America | Colombia |
| 15 | SRR1562524 |  | de Oliveira et al. 2017 | South America | Colombia |
| 16 | SRR1568221 |  | de Oliveira et al. 2017 | South America | Colombia |
| 17 | SRR1568236 |  | de Oliveira et al. 2017 | South America | Colombia |
| 18 | SRR1562967 |  | de Oliveira et al. 2017 | South America | Colombia |
| 19 | SRR1568171 |  | de Oliveira et al. 2017 | South America | Colombia |
| 20 | SRR1568155 |  | de Oliveira et al. 2017 | South America | Colombia |
| 21 | SRR1568227 |  | de Oliveira et al. 2017 | South America | Colombia |
| 22 | SRR1562975 |  | de Oliveira et al. 2017 | South America | Colombia |
| 23 | SRR1568230 |  | de Oliveira et al. 2017 | South America | Colombia |
| 24 | SRR1567977 |  | de Oliveira et al. 2017 | South America | Colombia |
| 25 | SRR1562971 |  | de Oliveira et al. 2017 | South America | Colombia |
| 26 | SRR1562555 |  | de Oliveira et al. 2017 | South America | Colombia |
| 27 | SRR1568207 |  | de Oliveira et al. 2017 | South America | Colombia |
| 28 | SRR1568118 |  | de Oliveira et al. 2017 | South America | Colombia |
| 29 | SRR1564670 |  | de Oliveira et al. 2017 | South America | Colombia |
| 30 | SRR1562870 |  | de Oliveira et al. 2017 | South America | Colombia |
| 31 | SRR1568213 |  | de Oliveira et al. 2017 | South America | Colombia |
| 32 | SRR1568160 |  | de Oliveira et al. 2017 | South America | Colombia |
| 33 | SRR1562518 |  | de Oliveira et al. 2017 | South America | Colombia |
| 34 | SRR1564665 |  | de Oliveira et al. 2017 | South America | Colombia |
| 35 | SRR1564660 |  | de Oliveira et al. 2017 | South America | Colombia |
| 36 | SRR1568169 |  | de Oliveira et al. 2017 | South America | Colombia |
| 37 | SRR1562965 |  | de Oliveira et al. 2017 | South America | Colombia |
| 38 | SRR1562818 |  | de Oliveira et al. 2017 | South America | Colombia |
| 48 | SRR1568121 | SRP046098 | Hupalo et al, 2016 | South America | Brazil |
| 40 | SRR1568116 | SRP046104 | Hupalo et al, 2016 | South America | Brazil |
| 39 | SRR1568197 | SRP046275 | Hupalo et al, 2016 | South America | Brazil |
| 41 | SRR1568176 | SRP046206 | Hupalo et al, 2016 | South America | Brazil |
| 42 | SRR1568108 | SRP046145 | Hupalo et al, 2016 | South America | Brazil |
| 43 | SRR1562973 | SRP046034 | Hupalo et al, 2016 | South America | Brazil |
| 44 | SRR1568233 | SRP046112 | Hupalo et al, 2016 | South America | Brazil |
| 45 | SRR1568173 | SRP046014 | Hupalo et al, 2016 | South America | Brazil |
| 61 |  | ERR3813239 | Ernest Diez Benavente et al. 2020 | South America | Brazil |
| 62 |  | ERR3813238 | Ernest Diez Benavente et al. 2020 | South America | Brazil |
| 63 |  | ERR3813237 | Ernest Diez Benavente et al. 2020 | South America | Brazil |
| 64 |  | ERR3813236 | Ernest Diez Benavente et al. 2020 | South America | Brazil |
| 65 |  | ERR3813235 | Ernest Diez Benavente et al. 2020 | South America | Brazil |
| 66 |  | ERR3813234 | Ernest Diez Benavente et al. 2020 | South America | Brazil |
| 67 |  | ERR3813233 | Ernest Diez Benavente et al. 2020 | South America | Brazil |
| 68 |  | ERR3813232 | Ernest Diez Benavente et al. 2020 | South America | Brazil |
| 69 |  | ERR3813231 | Ernest Diez Benavente et al. 2020 | South America | Brazil |
| 70 |  | ERR3813230 | Ernest Diez Benavente et al. 2020 | South America | Brazil |
| 71 |  | ERR3813229 | Ernest Diez Benavente et al. 2020 | South America | Brazil |
| 72 |  | ERR3813228 | Ernest Diez Benavente et al. 2020 | South America | Brazil |
| 73 |  | ERR3813227 | Ernest Diez Benavente et al. 2020 | South America | Brazil |
| 74 |  | ERR3813226 | Ernest Diez Benavente et al. 2020 | South America | Brazil |
| 75 |  | ERR3813225 | Ernest Diez Benavente et al. 2020 | South America | Brazil |
| 76 |  | ERR3813224 | Ernest Diez Benavente et al. 2020 | South America | Brazil |
| 46 | ERS564522 | ERR775190 | Auburn et al. 2019 | Africa | Ethiopia |
| 47 | ERS666260 | ERR925441 | Auburn et al. 2019 | Africa | Ethiopia |
| 77 | ERS849384 | ERR2678989 | Ford et al. 2020 | Africa | Ethiopia |
| 78 | ERS2593850 | ERR2678991 | Ford et al. 2020 | Africa | Ethiopia |
| 79 | ERS2593862 | ERR2679003 | Ford et al. 2020 | Africa | Ethiopia |
| 80 | ERS2593852 | ERR2678993 | Ford et al. 2020 | Africa | Ethiopia |
| 81 | ERS2593853 | ERR2678994 | Ford et al. 2020 | Africa | Ethiopia |
| 82 | ERS2593864 | ERR2679005 | Ford et al. 2020 | Africa | Ethiopia |
| 83 | ERS2593865 | ERR2679006 | Ford et al. 2020 | Africa | Ethiopia |
| 84 | ERS2593855 | ERR2678996 | Ford et al. 2020 | Africa | Ethiopia |
| 85 | ERS2593857 | ERR2678998 | Ford et al. 2020 | Africa | Ethiopia |
| 86 | ERS2593866 | ERR2679007 | Ford et al. 2020 | Africa | Ethiopia |
| 87 | ERS2593858 | ERR2678999 | Ford et al. 2020 | Africa | Ethiopia |
| 88 | ERS2593859 | ERR2679000 | Ford et al. 2020 | Africa | Ethiopia |
| 89 | ERS2593867 | ERR2679008 | Ford et al. 2020 | Africa | Ethiopia |
| 90 | ERS2593868 | ERR2679009 | Ford et al. 2020 | Africa | Ethiopia |
| 91 | ERS2593861 | ERR2679002 | Ford et al. 2020 | Africa | Ethiopia |
| 92 | ERS2593871 | ERR2679012 | Ford et al. 2020 | Africa | Ethiopia |
| 49 | SRR1562669 | SRP045995 | Hupalo et al, 2016 | Oceania | Papua New Guinea |
| 50 | SRR1562672 | SRP045997 | Hupalo et al, 2016 | Oceania | Papua New Guinea |
| 51 | SRR1562960 | SRP046023 | Hupalo et al, 2016 | Oceania | Papua New Guinea |
| 52 | SRR1562963 | SRP046026 | Hupalo et al, 2016 | Oceania | Papua New Guinea |
| 53 | SRR1568105 | SRP046095 | Hupalo et al, 2016 | Oceania | Papua New Guinea |
| 54 | SRR1568177 | SRP046188 | Hupalo et al, 2016 | Oceania | Papua New Guinea |
| 55 | SRR1568185 | SRP046136 | Hupalo et al, 2016 | Oceania | Papua New Guinea |
| 56 | SRR1568189 | SRP046141 | Hupalo et al, 2016 | Oceania | Papua New Guinea |
| 57 | SRR1568214 | SRP046176 | Hupalo et al, 2016 | Oceania | Papua New Guinea |
| 58 | SRR1759411 | SRP046126 | Hupalo et al, 2016 | Oceania | Papua New Guinea |
| 59 | SRR1759523 | SRP046126 | Hupalo et al, 2016 | Oceania | Papua New Guinea |
| 60 | SRR1759592 | SRP046126 | Hupalo et al, 2016 | Oceania | Papua New Guinea |
| 139 | ERS150822 | ERR175555 | Pearson et al, 2016 | Oceania | Papua New Guinea |
| 140 | ERS150862 | ERR175557 | Pearson et al, 2016 | Oceania | Papua New Guinea |
| 141 | ERS012699 | ERR022864 | Pearson et al, 2016 | Oceania | Papua New Guinea |
| 142 | ERS164683 | ERR216469 | Pearson et al, 2016 | Oceania | Papua New Guinea |
| 143 | ERS150819 | ERR175552 | Pearson et al, 2016 | Oceania | Papua New Guinea |
| 144 | ERS164688 | ERR216474 | Pearson et al, 2016 | Oceania | Papua New Guinea |
| 145 | ERS012057 | ERR020124 | Pearson et al, 2016 | Oceania | Papua New Guinea |
| 146 | ERS404141 | ERR527453 | Pearson et al, 2016 | Oceania | Papua New Guinea |
| 93 | ERS123115 | ERR152407 | Pearson et al, 2016 | South America | Brazil |
| 94 | ERS010153 | ERR018932 | Pearson et al, 2016 | South America | Brazil |
| 95 | ERS040108 | ERR054080 | Pearson et al, 2016 | Southeast Asia | Cambodia |
| 96 | ERS010154 | ERR020103 | Pearson et al, 2016 | Southeast Asia | Cambodia |
| 97 | ERS123116 | ERR152408 | Pearson et al, 2016 | Southeast Asia | Cambodia |
| 98 | ERS164689 | ERR216475 | Pearson et al, 2016 | Southeast Asia | Cambodia |
| 99 | ERS164670 | ERR211557 | Pearson et al, 2016 | Southeast Asia | Cambodia |
| 100 | ERS164692 | ERR216478 | Pearson et al, 2016 | Southeast Asia | Cambodia |
| 101 | ERS014174 | ERR023039 | Pearson et al, 2016 | Southeast Asia | Cambodia |
| 102 | ERS014175 | ERR023040 | Pearson et al, 2016 | Southeast Asia | Cambodia |
| 103 | ERS164662 | ERR211549 | Pearson et al, 2016 | Southeast Asia | Cambodia |
| 104 | ERS014179 | ERR027119 | Pearson et al, 2016 | Southeast Asia | Cambodia |
| 105 | ERS055897 | ERR111729 | Pearson et al, 2016 | Southeast Asia | Cambodia |
| 106 | ERS123117 | ERR152410 | Pearson et al, 2016 | Southeast Asia | Cambodia |
| 107 | ERS164691 | ERR216477 | Pearson et al, 2016 | Southeast Asia | Cambodia |
| 108 | ERS123119 | ERR152413 | Pearson et al, 2016 | Southeast Asia | Cambodia |
| 109 | ERS025405 | ERR039234 | Pearson et al, 2016 | Southeast Asia | Cambodia |
| 110 | ERS071835 | ERR123849 | Pearson et al, 2016 | Southeast Asia | Cambodia |
| 111 | ERS336352 | ERR388742 | Pearson et al, 2016 | Southeast Asia | Cambodia |
| 112 | ERS224881 | ERR337538 | Pearson et al, 2016 | Southeast Asia | Cambodia |
| 113 | ERS224901 | ERR337542 | Pearson et al, 2016 | Southeast Asia | Cambodia |
| 114 | ERS338595 | ERR386536 | Pearson et al, 2016 | Southeast Asia | Cambodia |
| 115 | ERS338597 | ERR386532 | Pearson et al, 2016 | Southeast Asia | Cambodia |
| 116 | ERS444642 | ERR586226 | Pearson et al, 2016 | Southeast Asia | Cambodia |
| 117 | ERS338598 | ERR386537 | Pearson et al, 2016 | Southeast Asia | Cambodia |
| 118 | ERS338599 | ERR386538 | Pearson et al, 2016 | Southeast Asia | Cambodia |
| 119 | ERS338601 | ERR386539 | Pearson et al, 2016 | Southeast Asia | Cambodia |
| 120 | ERS338602 | ERR386533 | Pearson et al, 2016 | Southeast Asia | Cambodia |
| 121 | ERS338603 | ERR386540 | Pearson et al, 2016 | Southeast Asia | Cambodia |
| 122 | ERS338605 | ERR386534 | Pearson et al, 2016 | Southeast Asia | Cambodia |
| 123 | ERS338607 | ERR386535 | Pearson et al, 2016 | Southeast Asia | Cambodia |
| 124 | ERS338610 | ERR386541 | Pearson et al, 2016 | Southeast Asia | Cambodia |
| 125 | ERS338612 | ERR386543 | Pearson et al, 2016 | Southeast Asia | Cambodia |
| 126 | ERS338617 | ERR386544 | Pearson et al, 2016 | Southeast Asia | Cambodia |
| 127 | ERS338618 | ERR386545 | Pearson et al, 2016 | Southeast Asia | Cambodia |
| 128 | ERS338619 | ERR386546 | Pearson et al, 2016 | Southeast Asia | Cambodia |
| 129 | ERS338621 | ERR386547 | Pearson et al, 2016 | Southeast Asia | Cambodia |
| 130 | ERS040113 | ERR054085 | Pearson et al, 2016 | Southeast Asia | Indonesia |
| 131 | ERS241435 | ERR337624 | Pearson et al, 2016 | Southeast Asia | Indonesia |
| 132 | ERS241441 | ERR337630 | Pearson et al, 2016 | Southeast Asia | Indonesia |
| 133 | ERS174628 | ERR221486 | Pearson et al, 2016 | Southeast Asia | Laos |
| 134 | ERS347479 | ERR426037 | Pearson et al, 2016 | Southeast Asia | Laos |
| 135 | ERS040116 | ERR054088 | Pearson et al, 2016 | Southeast Asia | Malaysia |
| 136 | ERS040117 | ERR054089 | Pearson et al, 2016 | Southeast Asia | Malaysia |
| 137 | ERS123120 | ERR152414 | Pearson et al, 2016 | Southeast Asia | Malaysia |
| 138 | ERS123124 | ERR152415 | Pearson et al, 2016 | Southeast Asia | Malaysia |
| 147 | ERS055892 | ERR111724 | Pearson et al, 2016 | Southeast Asia | Thailand |
| 148 | ERS055878 | ERR111710 | Pearson et al, 2016 | Southeast Asia | Thailand |
| 149 | ERS055895 | ERR111727 | Pearson et al, 2016 | Southeast Asia | Thailand |
| 150 | ERS055889 | ERR111721 | Pearson et al, 2016 | Southeast Asia | Thailand |
| 151 | ERS055896 | ERR111728 | Pearson et al, 2016 | Southeast Asia | Thailand |
| 152 | ERS055881 | ERR111713 | Pearson et al, 2016 | Southeast Asia | Thailand |
| 153 | ERS055887 | ERR111719 | Pearson et al, 2016 | Southeast Asia | Thailand |
| 154 | ERS055882 | ERR111714 | Pearson et al, 2016 | Southeast Asia | Thailand |
| 155 | ERS055893 | ERR111725 | Pearson et al, 2016 | Southeast Asia | Thailand |
| 156 | ERS055886 | ERR111718 | Pearson et al, 2016 | Southeast Asia | Thailand |
| 157 | ERS055883 | ERR111715 | Pearson et al, 2016 | Southeast Asia | Thailand |
| 158 | ERS055891 | ERR111723 | Pearson et al, 2016 | Southeast Asia | Thailand |
| 159 | ERS055890 | ERR111722 | Pearson et al, 2016 | Southeast Asia | Thailand |
| 160 | ERS055880 | ERR111712 | Pearson et al, 2016 | Southeast Asia | Thailand |
| 161 | ERS055879 | ERR111711 | Pearson et al, 2016 | Southeast Asia | Thailand |
| 162 | ERS142861 | ERR164695 | Pearson et al, 2016 | Southeast Asia | Thailand |
| 163 | ERS241409 | ERR337599 | Pearson et al, 2016 | Southeast Asia | Thailand |
| 164 | ERS241418 | ERR337608 | Pearson et al, 2016 | Southeast Asia | Thailand |
| 165 | ERS241427 | ERR337616 | Pearson et al, 2016 | Southeast Asia | Thailand |
| 166 | ERS241430 | ERR337619 | Pearson et al, 2016 | Southeast Asia | Thailand |
| 167 | ERS241433 | ERR337622 | Pearson et al, 2016 | Southeast Asia | Thailand |
| 168 | ERS241439 | ERR337628 | Pearson et al, 2016 | Southeast Asia | Thailand |
| 169 | ERS241406 | ERR337596 | Pearson et al, 2016 | Southeast Asia | Thailand |
| 170 | ERS241410 | ERR337600 | Pearson et al, 2016 | Southeast Asia | Thailand |
| 171 | ERS241416 | ERR337606 | Pearson et al, 2016 | Southeast Asia | Thailand |
| 172 | ERS241422 | ERR337611 | Pearson et al, 2016 | Southeast Asia | Thailand |
| 173 | ERS241428 | ERR337617 | Pearson et al, 2016 | Southeast Asia | Thailand |
| 174 | ERS241437 | ERR337626 | Pearson et al, 2016 | Southeast Asia | Thailand |
| 175 | ERS241407 | ERR337597 | Pearson et al, 2016 | Southeast Asia | Thailand |
| 176 | ERS241411 | ERR337601 | Pearson et al, 2016 | Southeast Asia | Thailand |
| 177 | ERS241417 | ERR337607 | Pearson et al, 2016 | Southeast Asia | Thailand |
| 178 | ERS241429 | ERR337618 | Pearson et al, 2016 | Southeast Asia | Thailand |
| 179 | ERS241432 | ERR337621 | Pearson et al, 2016 | Southeast Asia | Thailand |
| 180 | ERS347497 | ERR426015 | Pearson et al, 2016 | Southeast Asia | Thailand |
| 181 | ERS403538 | ERR527355 | Pearson et al, 2016 | Southeast Asia | Thailand |
| 182 | ERS403542 | ERR527356 | Pearson et al, 2016 | Southeast Asia | Thailand |
| 183 | ERS403554 | ERR527370 | Pearson et al, 2016 | Southeast Asia | Thailand |
| 184 | ERS403535 | ERR527366 | Pearson et al, 2016 | Southeast Asia | Thailand |
| 185 | ERS403539 | ERR527378 | Pearson et al, 2016 | Southeast Asia | Thailand |
| 186 | ERS403543 | ERR527367 | Pearson et al, 2016 | Southeast Asia | Thailand |
| 187 | ERS403559 | ERR527372 | Pearson et al, 2016 | Southeast Asia | Thailand |
| 188 | ERS403563 | ERR527384 | Pearson et al, 2016 | Southeast Asia | Thailand |
| 189 | ERS403528 | ERR527377 | Pearson et al, 2016 | Southeast Asia | Thailand |
| 190 | ERS403532 | ERR527340 | Pearson et al, 2016 | Southeast Asia | Thailand |
| 191 | ERS403556 | ERR527348 | Pearson et al, 2016 | Southeast Asia | Thailand |
| 192 | ERS403529 | ERR527339 | Pearson et al, 2016 | Southeast Asia | Thailand |
| 193 | ERS403545 | ERR527368 | Pearson et al, 2016 | Southeast Asia | Thailand |
| 194 | ERS403549 | ERR527346 | Pearson et al, 2016 | Southeast Asia | Thailand |
| 195 | ERS013104 | ERR023161 | Pearson et al, 2016 | Southeast Asia | Vietnam |
| 196 | ERS012702 | ERR022866 | Pearson et al, 2016 | Southeast Asia | Vietnam |
| 197 | ERS164634 | ERR484718 | Pearson et al, 2016 | Southeast Asia | Vietnam |
| 198 | ERS012693 | ERR021981 | Pearson et al, 2016 | Southeast Asia | Vietnam |
| 199 | ERS012695 | ERR021983 | Pearson et al, 2016 | Southeast Asia | Vietnam |
| 200 | ERS012694 | ERR021982 | Pearson et al, 2016 | Southeast Asia | Vietnam |
| 201 | ERS164639 | ERR211538 | Pearson et al, 2016 | Southeast Asia | Vietnam |
| 202 | ERS164684 | ERR216470 | Pearson et al, 2016 | Southeast Asia | Vietnam |
| 203 | ERS164678 | ERR211565 | Pearson et al, 2016 | Southeast Asia | Vietnam |
| 204 | ERS336392 | ERR388796 | Pearson et al, 2016 | Southeast Asia | Vietnam |
| 205 | ERS143422 | ERR175494 | Pearson et al, 2016 | Southeast Asia | Vietnam |
| 206 |  | SRR10969353 | Brashear et al, 2020 | Southeast Asia | China-Myanmar border |
| 207 |  | SRR10969355 | Brashear et al, 2020 | Southeast Asia | China-Myanmar border |
| 208 |  | SRR10969356 | Brashear et al, 2020 | Southeast Asia | China-Myanmar border |
| 209 |  | SRR10969357 | Brashear et al, 2020 | Southeast Asia | China-Myanmar border |
| 210 |  | SRR10969358 | Brashear et al, 2020 | Southeast Asia | China-Myanmar border |
| 211 |  | SRR10969359 | Brashear et al, 2020 | Southeast Asia | China-Myanmar border |
| 212 |  | SRR10969361 | Brashear et al, 2020 | Southeast Asia | China-Myanmar border |
| 213 |  | SRR10969362 | Brashear et al, 2020 | Southeast Asia | China-Myanmar border |
| 214 |  | SRR10969363 | Brashear et al, 2020 | Southeast Asia | China-Myanmar border |
| 215 |  | SRR10969364 | Brashear et al, 2020 | Southeast Asia | China-Myanmar border |
| 216 |  | SRR10969365 | Brashear et al, 2020 | Southeast Asia | China-Myanmar border |
| 217 |  | SRR10969366 | Brashear et al, 2020 | Southeast Asia | China-Myanmar border |
| 218 |  | SRR10969368 | Brashear et al, 2020 | Southeast Asia | China-Myanmar border |
| 219 |  | SRR10969369 | Brashear et al, 2020 | Southeast Asia | China-Myanmar border |
| 220 |  | SRR10969370 | Brashear et al, 2020 | Southeast Asia | China-Myanmar border |
| 221 |  | SRR10969371 | Brashear et al, 2020 | Southeast Asia | China-Myanmar border |
| 222 |  | SRR10969372 | Brashear et al, 2020 | Southeast Asia | China-Myanmar border |
| 223 |  | SRR10969373 | Brashear et al, 2020 | Southeast Asia | China-Myanmar border |
| 224 |  | SRR10969375 | Brashear et al, 2020 | Southeast Asia | China-Myanmar border |

Table S3. Genetic mutations of *PvRBP2b* near-full length in different areas.

| Mutations at nucleotide | Populations | | | | | | | | | | | | Mutations at amino acid |
| --- | --- | --- | --- | --- | --- | --- | --- | --- | --- | --- | --- | --- | --- |
|  | Cam (n=35) | Vie (n=11) | Mal (n=4) | Indo (n=3) | Laos(n=2) | PNG (n=20) | Bra (n=36) | Col(n=28) | Eth (n=18) | CM (n=63) | Tha (n=92) | Total (n=312) |  |
| **G406A** | 30(85.7%) | 9(81.8%) | 3(75.0%) | 3(100.0%) | 2(100.0%) | 18(90.0%) | 7(19.4%) | 1(3.6%) | 5(27.8%) | 26(41.3%) | 76(82.6%) | 180(57.7%) | **E136K** |
| **G415C** |  |  |  |  |  |  |  |  |  | 12(19.0%) |  | 12(3.9%) | **E139Q** |
| G620T |  |  |  |  |  |  |  |  |  |  | 3(3.3%) | 3(1.0%) | R207L |
| **G650A** | 12(34.3%) | 2(18.2%) | 1(25.0%) |  |  | 1(5.0%) |  |  | 1(5.6%) | 5(7.9%) | 19(20.7%) | 41(13.1%) | **R217H** |
| **G658T** |  |  |  |  |  |  | 6(16.6%) | 12(42.9%) | 4(22.2%) | 1(1.6%) |  | 23(7.4%) | **D220Y** |
| **C671A/G** | 12(34.3%) | 3(27.3%) | 1(25.0%) | 2(66.7%) |  | 9(45.0%) |  |  |  | 6(9.5%) | 32(34.8%) | 65(20.8%) | **T224R/K** |
| **T682C** |  |  |  |  |  |  |  |  |  | 3(4.8%) | 2(2.2%) | 5(1.6%) | **S228P** |
| C682T |  |  |  |  |  |  |  |  |  |  | 2(2.2%) | 2(0.6%) | L229F/V |
| C693T |  |  |  |  |  |  |  |  |  |  | 1(1.1%) | 1(0.3%) | N231K |
| **G694A** | 4(11.4%) | 2(18.2%) | 1(25.0%) |  |  |  |  |  |  | 3(4.8%) | 6(6.5%) | 16(5.1%) | **E232K** |
| **G725C** | 5(14.3%) | 2(18.2%) | 1(25.0%) |  | 1(50.0%) |  |  |  |  | 3(4.8%) | 7(7.6%) | 19(6.1%) | **R242T/S** |
| **G726C** | 20(57.1%) |  | 1(25.0%) |  |  |  | 16(44.4%) | 9(32.1%) | 9(50.0%) | 19(30.2%) | 42(45.7%) | 116(37.2%) |  |
| C852G |  |  |  |  |  |  |  |  |  |  | 2(2.2%) | 2(0.6%) | C284W |
| **A862G** | 6(17.1%) | 3(27.3%) | 1(25.0%) |  |  |  | 7(19.4%) | 1(3.6%) |  | 20(31.7%) | 28(30.4%) | 66(21.2%) | **K288E/T** |
| **A863C** | 5(14.3%) | 2(18.2%) |  |  |  | 1(5.0%) | 1(2.7%) |  | 4(22.2%) |  | 8(8.7%) | 21(6.7%) |  |
| **T877G** |  |  |  |  |  |  | 1(2.7%) | 6(21.4%) | 4(22.2%) |  |  | 11(3.5%) | **L293V** |
| A894T |  |  |  |  |  |  |  |  |  |  | 2(2.2%) | 2(0.6%) | K298N |
| **A898G** |  |  |  |  |  |  |  | 5(17.9%) |  |  |  | 5(1.6%) | **N300K/D** |
| **T900A** |  |  |  |  |  |  | 1(2.7%) | 6(21.4%) | 4(22.2%) |  |  | 11(3.5%) |  |
| **A925C** | 6(17.1%) | 2(18.2%) | 1(25.0%) |  | 1(50.0%) |  |  |  |  | 3(4.8%) | 14(15.2%) | 27(8.6%) | **K309Q** |
| **G943T** | 6(17.1%) | 2(18.2%) | 1(25.0%) |  | 1(50.0%) | 1(5.0%) |  |  |  | 2(3.2%) | 11(12.0%) | 24(7.7%) | **D315Y** |
| G1012A |  |  |  |  |  |  |  |  |  |  | 1(1.1%) | 1(0.3%) | E338K |
| **T1047A** | 22(62.9%) | 5(45.5%) | 3(75.0%) | 3(100.0%) | 2(100.0%) | 6(30.0%) | 15(41.6%) | 15(53.6%) | 10(55.6%) | 8(12.7%) | 37(40.2%) | 126(40.4%) | **N349K** |
| **A1087G** | 21(60.0%) | 5(45.5%) | 1(25.0%) | 1(33.3%) | 2(100.0%) | 11(55.0%) | 17(47.2%) | 15(53.6%) | 11(61.1%) | 8(12.7%) | 34(37.0%) | 126(40.4%) | **K363E** |
| **G1096C** | 5(14.3%) | 1(9.1%) | 1(25.0%) | 2(66.7%) |  | 5(25.0%) |  |  |  |  | 5(5.4%) | 19(6.1%) | **D366V/H** |
| **A1097T** | 15(42.9%) | 5(45.5%) | 2(50.0%) | 1(33.3%) | 2(100.0%) | 2(10.0%) | 16(44.4%) | 13(46.4%) | 10(55.6%) | 8(12.7%) | 35(38.0%) | 109(34.9%) |  |
| **C1098T** | 16(45.7%) | 4(36.4%) | 2(50.0%) | 1(33.3%) | 2(100.0%) | 2(10.0%) | 17(47.2%) | 13(46.4%) | 10(55.6%) | 8(12.7%) | 35(38.0%) | 110(35.3%) |  |
| **G1144C/A** | 5(14.3%) | 3(27.3%) |  |  |  |  |  |  |  | 1(1.6%) | 5(5.4%) | 14(4.5%) | **G382R/E** |
| **G1145A** | 5(14.3%) | 1(9.1%) |  |  |  | 3(15.0%) | 10(27.8%) | 1(3.6%) | 11(61.1%) | 20(31.7%) | 32(34.8%) | 83(26.6%) |  |
| **G1183A** |  |  |  |  |  |  |  |  |  |  | 3(3.3%) | 3(1.0%) | **V395A/T** |
| **T1184C** | 22(62.9%) | 7(63.6%) | 2(50.0%) | 2(66.7%) | 1(50.0%) | 18(90.0%) |  |  | 2(11.1%) | 9(14.3%) | 31(33.7%) | 94(30.1%) |  |
| **A1236T** | 18(51.4%) | 6(54.5%) | 2(50.0%) |  |  | 1(5.0%) | 20(55.6%) | 9(32.1%) | 6(33.3%) | 32(50.8%) | 66(71.7%) | 160(51.3%) | **K412N** |
| **C1237G** | 5(14.3%) | 1(9.1%) | 1(25.0%) | 3(100.0%) |  | 16(80.0%) |  |  | 1(5.6%) |  | 6(6.5%) | 33(10.6%) | **Q413E** |
| **A1309G** | 1(2.9%) |  | 1(25.0%) | 1(33.3%) |  | 1(5.0%) |  |  |  |  | 3(3.3%) | 7(2.2%) | **K437E** |
| G1351A |  |  |  |  |  |  | 3(8.3%) |  |  |  |  | 3(1.0%) | E451K |
| **C1365A** | 4(11.4%) | 2(18.2%) | 1(25.0%) |  |  |  |  |  |  | 5(7.9%) | 7(7.6%) | 19(6.1%) | **H455Q** |
| **G1489A** | 5(14.3%) | 2(18.2%) | 1(25.0%) |  | 1(50.0%) | 2(10.0%) |  |  |  | 5(7.9%) | 11(12.0%) | 27(8.7%) | **E497K** |
| **C1674A** | 6(17.1%) | 3(27.3%) | 2(50.0%) |  |  |  |  |  |  | 3(4.8%) | 5(5.4%) | 19(6.1%) | **D558E** |
| **A1691G** | 17(48.6%) | 6(54.5%) | 4(100.0%) | 2(66.7%) | 1(50.0%) | 11(55.0%) | 10(27.8%) | 1(3.6%) | 4(22.2%) | 21(33.3%) | 58(63.0%) | 135(43.3%) | **Q564R** |
| **A1723G** | 1(2.9%) |  |  |  |  | 1(5.0%) | 10(27.8%) |  | 2(11.1%) |  | 5(5.4%) | 19(6.1%) | **K575E** |
| **G1732C** |  |  |  | 1(33.3%) |  | 6(30.0%) |  |  |  |  |  | 7(2.2%) | **D578H** |
| **C1758A** |  |  |  |  |  |  | 8(22.2%) |  | 2(11.1%) |  | 1(1.1%) | 11(3.5%) | **S586R** |
| **T1773A** | 7(20.0%) |  |  | 1(33.3%) |  | 6(30.0%) | 7(19.4%) | 2(7.1%) | 1(5.6%) | 10(15.9%) | 29(31.5%) | 63(20.2%) | **N591K** |
| **G1891A** |  |  | 1(25.0%) | 1(33.3%) |  | 1(5.0%) |  |  |  |  | 5(5.4%) | 8(2.6%) | **E631K** |
| **C1997A** |  |  |  |  |  | 5(25.0%) |  |  |  |  |  | 5(1.6%) | **A666E** |
| G2468A |  |  |  |  |  |  |  |  | 3(16.7%) |  |  | 3(1.0%) | G823E |
| **C2751A** | 34(97.1%) | 11(100.0%) | 4(100.0%) | 3(100.0%) | 2(100.0%) | 18(90.0%) | 27(75%) | 27(96.4%) | 18(100.0%) | 63(100.0%) | 91(98.9%) | 298(95.5%) | **D917E** |
| **G2973A** | 2(5.7%) | 1(9.1%) |  |  |  |  | 1(2.8%) |  | 1(5.6%) |  |  | 5(1.6%) | **M991I** |
| G3344A |  |  |  |  |  |  |  |  |  | 1(1.6%) |  | 1(0.3%) | G1115E |
| G3349A |  |  |  |  |  |  |  |  | 3(16.7%) |  |  | 3(1.0%) | D1117N |
| C3502A |  |  |  |  |  |  |  |  |  |  | 2(2.2%) | 2(0.6%) | L1168I |
| **A3518G** |  |  |  |  |  | 4(20.0%) |  |  |  |  |  | 4(1.3%) | **Q1173R** |
| **T3542C** | 1(2.9%) | 3(27.3%) |  |  |  |  |  |  |  |  | 5(5.4%) | 9(2.9%) | **V1181A** |
| A3661G |  |  |  |  |  |  |  |  |  | 1(1.6%) |  | 1(0.3%) | K1221E |
| **A3716G** | 1(2.9%) |  | 1(25.0%) | 2(66.7%) |  | 1(5.0%) |  |  |  |  | 9(9.8%) | 14(4.5%) | **E1239G** |
| G3859A |  |  |  |  |  |  |  |  |  |  | 1(1.1%) | 1(0.3%) | E1287K |
| **T3866C** | 1(2.9%) |  |  |  |  |  |  |  |  |  | 5(5.4%) | 6(1.9%) | **V1289A** |
| G3938C |  |  |  |  |  |  |  |  |  | 2(3.2%) |  | 2(0.6%) | S1313T |
| A4054G |  |  |  |  |  |  | 3(8.3%) |  |  |  |  | 3(1.0%) | T1352A |
| **C4142A** | 2(5.7%) | 1(9.1%) |  |  |  |  |  |  |  | 2(3.2%) | 11(12.0%) | 16(5.1%) | **T1381N** |
| **G4528A** | 2(5.7%) | 1(9.1%) |  | 1(33.3%) |  |  |  |  |  | 3(4.8%) | 7(7.6%) | 14(4.5%) | **V1510I** |
| **C4558T** |  |  |  | 0.00% |  |  | 4(11.1%) | 8(28.6%) | 1(5.6%) |  |  | 13(4.2%) | **H1520Y** |
| **C4587A** | 13(37.1%) | 5(45.5%) | 1(25.0%) | 3(100.0%) | 1(50.0%) | 19(95.0%) | 16(44.4%) | 1(3.6%) | 3(16.7%) | 32(50.8%) | 35(38.0%) | 129(41.4%) | **N1529K** |
| T4632G |  |  |  |  |  |  |  |  |  | 2(3.2%) |  | 2(0.6%) | D1544E |
| **G4729A** |  |  |  | 1(33.3%) |  | 2(10.0%) | 1(2.8%) |  |  |  |  | 4(1.3%) | **G1577R** |
| **A4816G** | 29(82.9%) | 10(90.9%) | 4(100.0%) | 3(100.0%) | 2(100.0%) | 19(95.0%) | 1(2.8%) | 9(32.1%) | 11(61.1%) | 48(76.2%) | 82(89.1%) | 237(76.0%) | **K1606E** |
| **A5104G** |  |  |  |  |  |  |  |  |  | 25(39.7%) |  | 25(8.0%) | **I1702V** |
| **A5462C** |  |  |  |  |  |  |  |  |  | 1(1.6%) | 3(3.3%) | 4(1.3%) | **K1821T** |
| **G5609A** | 2(5.7%) |  |  |  |  |  |  |  |  | 4(6.3%) | 5(5.4%) | 11(3.5%) | **G1870D** |
| **A5720C** | 1(2.9%) |  |  |  |  |  |  |  |  |  | 6(6.5%) | 7(2.2%) | **K1907T** |
| **T5951G** |  |  |  |  |  |  |  |  |  |  | 5(5.4%) | 5(1.6%) | **V1984G** |
| **C5977G** | 1(2.9%) |  |  | 1(33.3%) |  | 2(10.0%) |  |  |  |  |  | 4(1.3%) | **Q1993E** |
| G6023A |  |  |  |  |  |  |  |  |  | 1(1.6%) |  | 1(0.3%) | C2008Y |
| **C6218A** | 2(5.7%) | 4(36.4%) |  |  | 1(50.0%) | 1(5.0%) |  |  |  | 32(50.8%) | 10(10.9%) | 50(16.0%) | **P2073H** |
| A6433C |  |  |  |  |  |  |  |  |  | 1(1.6%) |  | 1(0.3%) | K2145Q |
| **C6569T** |  |  |  |  |  |  |  |  |  | 30(47.6%) | 11(12.0%) | 41(13.1%) | **S2190F** |
| **G6598A** | 7(20.0%) |  |  |  |  |  |  |  |  |  | 5(5.4%) | 12(3.9%) | **A2200T** |
| **A6601C** |  |  |  |  |  |  |  |  |  |  | 5(5.4%) | 5(1.6%) | **T2201P** |
| **C6661A/G** | 5(14.3%) | 4(36.4%) |  |  | 1(50.0%) |  |  |  |  | 2(3.2%) | 17(18.5%) | 29(9.3%) | **L2221I** |
| A6683G |  |  |  |  |  |  |  |  |  | 1(1.6%) |  | 1(0.3%) | K2228R |
| **C6707G** |  |  |  |  |  |  |  |  |  | 10(15.9%) | 6(6.5%) | 16(5.1%) | **A2236G** |
| **G6748A/C** | 7(20.0%) | 1(9.1%) | 1(25.0%) | 1(33.3%) |  | 3(15.0%) | 15(41.7%) | 5(17.9%) | 4(22.2%) | 6(9.5%) | 19(20.7%) | 62(19.9%) | **A2250T/P** |
| A6779G |  |  |  |  |  |  |  |  |  | 3(4.8%) |  | 3(1.0%) | N2260S |
| **C6782T** |  |  |  |  |  |  |  |  |  | 40(63.5%) | 26(28.3%) | 66(21.2%) | **S2261F** |
| **G6793A** | 31(88.6%) | 9(81.8%) | 4(100.0%) | 3(100.0%) | 2(100.0%) | 17(85.0%) | 5(13.9%) |  | 4(22.2%) | 37(58.7%) | 86(93.5%) | 198(63.5%) | **E2265K** |
| **A6815C** |  |  |  |  |  |  |  |  |  |  | 6(6.5%) | 6(1.9%) | **H2272P** |
| **C6832T** |  |  |  |  |  |  |  |  |  | 6(9.5%) | 25(27.2%) | 31(9.9%) | **L2278F** |
| A6949C |  |  |  |  |  |  |  |  |  |  | 2(2.2%) | 2(0.6%) | T2317P |
| **C6953T** |  |  |  |  |  |  |  |  |  | 44(69.8%) | 30(32.6%) | 74(23.7%) | **S2318F** |
| T7109G |  |  |  |  |  |  |  |  |  |  | 3(3.3%) | 3(1.0%) | Y2370G |
| A7144T |  |  |  |  |  |  |  |  |  |  | 2(2.2%) | 2(0.6%) | T2382F |
| T7266G |  |  |  |  |  |  |  |  |  |  | 1(1.1%) | 1(0.3%) | N2422K |
| G7375A |  |  |  |  |  |  |  |  |  |  | 3(3.3%) | 3(1.0%) | E2459K |
| C7551G |  |  |  |  |  | 2(10%) |  |  |  |  | 1(1.1%) | 3(1.0%) | N2517K |
| G7643A |  |  |  |  |  |  |  |  |  |  | 1(1.1%) | 1(0.3%) | R2548I |
| **A7838T** |  |  |  |  |  |  |  |  |  | 12(19.0%) | 1(1.1%) | 13(4.2%) | **K2613I** |
| **G7925A** | 4(11.4%) |  |  |  |  |  |  |  |  | 3(4.8%) | 2(2.2%) | 9(2.9%) | **R2642K** |
| G8170C |  |  |  |  |  |  |  |  |  | 2(3.2%) |  | 2(0.6%) | E2724Q |
| **A8212G** | 2(5.7%) | 2(18.2%) | 1(25.0%) | 3(100.0%) |  | 14(70.0%) |  |  |  |  |  | 22(7.1%) | **I2738V** |
| **G8222C** | 4(11.4%) |  | 1(25.0%) |  |  | 3(15.0%) | 3(8.3%) | 4(14.3%) | 1(5.6%) |  | 3(3.3%) | 19(6.1%) | **R2741P** |
| **A8237G** | 6(17.1%) | 2(18.2%) | 2(50.0%) | 3(100.0%) |  | 19(95.0%) | 3(8.3%) | 5(17.9%) | 1(5.6%) | 25(39.7%) | 4(4.3%) | 70(22.4%) | **E2746G** |
| **G8240A** | 2(5.7%) | 2(18.2%) | 1(25.0%) | 3(100.0%) |  | 16(80.0%) |  |  |  |  |  | 24(7.7%) | **G2747E** |
| **G8250T** | 6(17.1%) | 2(18.2%) | 1(25.0%) | 3(100.0%) |  | 19(95.0%) |  |  | 1(5.6%) |  | 1(1.1%) | 33(10.6%) | **L2750F** |

Cam: Cambodia, Vie: Vietnam, Mal: Malaysia, Indo: Indonesia, Laos: Laos, PNG: Papua New Guinea, Bra: Brazil, Col: Colombia, Eth: Ethiopia, CM: China Myanmar, Tha: Thailand.

Table S4. Recombination events detected in the near full length of *PvRBP2b* gene by RDP4 package

| No | Recombinant | Major Parent | Minor Parent | Detection methods | |
| --- | --- | --- | --- | --- | --- |
| 1 | T2b-6 | Unknown | T2b-26 | R,G,M,C,S,3S | |
| 2 | C2b-1 | C2b-35 | C2b-27 | M,3S | |
| 3 | ERS012695 | T2b-5 | ERR216470 | M | |
| 4 | C2b-27 | Unknown | ERR216470 | M,S,3S | |
| 5 | C2b-3 | SRR5278301 | Unknown | 3S | |
| * R, RDP; G, GENECONV; M, MAXCHI; C, CHIMARERA; S, SISCAN; 3S,3SEQ | | | |  |  |

Table S5. Sequences and distribution of *PvRBP2b* reticulocyte binding region haplotypes

| Haplotype | | Populations ( N ) | | | | | | | | | |  | Total | Prevalence (%) |
| --- | --- | --- | --- | --- | --- | --- | --- | --- | --- | --- | --- | --- | --- | --- |
|  |  | CM | Tha | Cam | Vie | Mal | Ind | Lao | PNG | Eth | Bra | Col |  |  |
| Hap_1 | GCGAAATAACGTACAGT |  |  |  |  |  |  |  |  |  | 5 |  | 5 | 1.60 |
| Hap_2 | GCCAAATAACGTACAAT |  |  |  |  |  |  |  |  |  | 1 |  | 1 | 0.32 |
| Hap_3 | GCGAAATGTTGTTCAAT |  |  |  |  |  |  |  |  |  | 2 |  | 2 | 0.64 |
| Hap_4 | GCGAAAAGTTGTTCAAT |  |  |  |  |  |  |  |  | 3 | 2 | 2 | 7 | 2.24 |
| Hap_5 | GCCAAAAGTTGTTCAGT |  |  |  |  |  |  |  |  |  | 5 |  | 5 | 1.60 |
| Hap_6 | GCCGAATAACATTCGAT | 16 | 18 | 4 |  |  |  |  |  |  | 7 | 1 | 46 | 14.74 |
| Hap_7 | GCGAAATAACGTTCAAT | 1 |  | 1 |  |  |  |  |  | 2 | 1 |  | 5 | 1.60 |
| Hap_8 | GCGAAATAACGTACAAT | 25 | 1 |  |  |  |  |  |  | 1 | 1 | 12 | 40 | 12.82 |
| Hap_9 | GCCAAAAGACGTACAAT |  |  |  |  |  |  |  |  |  |  | 2 | 2 | 0.64 |
| Hap_10 | GCCAAAAGTTGTTCAAA |  |  |  |  |  |  |  |  |  |  | 2 | 2 | 0.64 |
| Hap_11 | GCGAAAAGTTGTACAAT | 1 |  |  |  |  |  |  |  | 1 |  | 5 | 7 | 2.24 |
| Hap_12 | GCCAAAAGTTGTTCAAT |  |  |  |  |  |  |  |  |  |  | 4 | 4 | 1.28 |
| Hap_13 | GCGACAAAACGTACAAT |  |  |  |  |  |  |  |  |  | 1 |  | 1 | 0.32 |
| Hap_14 | GCGAAATGATGTACAAT |  |  |  |  |  |  |  |  |  | 1 |  | 1 | 0.32 |
| Hap_15 | GCGAAAAGTTGTACAAA |  |  |  |  |  |  |  |  |  | 7 |  | 7 | 2.24 |
| Hap_16 | GCCAAATAACATTCGAT |  |  |  |  |  |  |  |  |  | 3 |  | 3 | 0.96 |
| Hap_17 | GCCACATAACGTACGAT |  |  |  |  |  |  |  |  | 3 |  |  | 3 | 0.96 |
| Hap_18 | GCCAAAAGTTGTACGAT |  |  |  |  |  |  |  |  | 1 |  |  | 1 | 0.32 |
| Hap_19 | GCCAAAAGTTGTACAAT |  |  |  |  |  |  |  |  | 2 |  |  | 2 | 0.64 |
| Hap_20 | GCCACATAACGTACAAT |  |  |  |  |  |  |  |  | 1 |  |  | 1 | 0.32 |
| Hap_21 | GCGAAAAGTTGCACAAT |  |  |  |  |  |  |  |  | 1 |  |  | 1 | 0.32 |
| Hap_22 | GCCAAAAGTTGTACAGT |  |  |  |  |  |  |  |  | 1 |  |  | 1 | 0.32 |
| Hap_23 | GCGAAAAGTTGTACAGT |  |  |  |  |  |  |  |  | 1 |  |  | 1 | 0.32 |
| Hap_24 | GCGAAATGACGCACAAA |  |  |  |  |  |  |  | 1 |  |  |  | 1 | 0.32 |
| Hap_25 | GCGAAATGACGCAGAAT |  |  |  |  |  |  |  | 3 |  |  |  | 3 | 0.96 |
| Hap_26 | GCGAAATGACGCAGAAA |  |  |  |  |  |  |  | 1 |  |  |  | 1 | 0.32 |
| Hap_27 | GCGAAAAAACGCAGGAT |  | 1 |  |  |  |  |  | 1 |  |  |  | 2 | 0.64 |
| Hap_28 | GAGAAAAAACGCAGGAT |  | 1 | 1 |  | 1 | 1 |  | 3 |  |  |  | 7 | 2.24 |
| Hap_29 | GAGAAATAACACAGGAT |  |  |  |  |  |  |  | 2 |  |  |  | 2 | 0.64 |
| Hap_30 | GCGAAATGACGCAGGAT |  |  |  |  |  |  |  | 2 |  |  |  | 2 | 0.64 |
| Hap_31 | GAGAAATAACACAGGAA |  |  |  |  |  |  |  | 1 |  |  |  | 1 | 0.32 |
| Hap_32 | GCGACATGACGCAGAAT |  |  |  |  |  |  |  | 1 |  |  |  | 1 | 0.32 |
| Hap_33 | GAGAAATAACGTAGGAA |  |  |  |  |  |  |  | 1 |  |  |  | 1 | 0.32 |
| Hap_34 | AAGAAAAAACGCAGAAA |  |  |  |  |  |  |  | 1 |  |  |  | 1 | 0.32 |
| Hap_35 | GCGAAAAGTTGCACAGT |  |  |  |  |  |  |  | 1 |  |  |  | 1 | 0.32 |
| Hap_36 | GAGAAATGTTGCTCGAT |  |  |  |  |  |  |  | 1 |  |  |  | 1 | 0.32 |
| Hap_37 | GCGAAATGACGTACAAA |  |  |  |  |  |  |  | 1 |  |  |  | 1 | 0.32 |
| Hap_38 | ACCAAATGACGCAGAAA |  |  |  |  |  |  |  |  | 1 |  |  | 1 | 0.32 |
| Hap_39 | GCGAACAGTTGCACGAT |  | 3 | 3 | 1 | 1 |  |  |  |  |  |  | 8 | 2.56 |
| Hap_40 | ACCACATAACGTACGAT |  | 1 |  | 1 |  |  |  |  |  |  |  | 2 | 0.64 |
| Hap_41 | GGGAAATGTTGTTCAAA |  |  |  | 1 |  |  |  |  |  |  |  | 1 | 0.32 |
| Hap_42 | ACGAAATAACGCTCAAA |  |  |  | 1 |  |  |  |  |  |  |  | 1 | 0.32 |
| Hap_43 | GCGAACAGTCGCACGAT |  |  |  | 1 |  |  |  |  |  |  |  | 1 | 0.32 |
| Hap_44 | GCCGAATAACGTTCGAT |  | 2 |  | 1 | 1 |  |  |  |  |  |  | 4 | 1.28 |
| Hap_45 | GCCACAAGTTGCTCGAA |  |  |  | 1 |  |  |  |  |  |  |  | 1 | 0.32 |
| Hap_46 | GACGAAAGTTGCTCAAA |  |  |  | 1 |  |  |  |  |  |  |  | 1 | 0.32 |
| Hap_47 | GCCGAATAACATTCAAA | 1 | 2 |  | 1 |  |  |  |  |  |  |  | 4 | 1.28 |
| Hap_48 | GCGAAATAACGCACAAA |  |  |  | 1 |  |  |  |  |  |  |  | 1 | 0.32 |
| Hap_49 | GAGAAAAAACGCAGGAA |  |  |  | 1 |  |  |  |  |  |  |  | 1 | 0.32 |
| Hap_50 | ACGAAAAATTGTTCGAT |  |  |  |  | 1 |  |  |  |  |  |  | 1 | 0.32 |
| Hap_51 | GCGAAAAGTTGTACGAT |  |  |  |  |  |  | 1 |  |  |  |  | 1 | 0.32 |
| Hap_52 | GCGAACAGTTGCACAAT | 3 | 3 |  |  |  |  | 1 |  |  |  |  | 7 | 2.24 |
| Hap_53 | GCGAACTAACGTTCGGT |  | 1 |  |  |  |  |  |  |  |  |  | 1 | 0.32 |
| Hap_54 | GAGAACTAACATTCGAT |  | 2 |  |  |  |  |  |  |  |  |  | 2 | 0.64 |
| Hap_55 | ACGAAATAACATTCGAT | 1 | 2 |  |  |  |  |  |  |  |  |  | 3 | 0.96 |
| Hap_56 | GAGAACTAACGTACGAT |  | 1 |  |  |  |  |  |  |  |  |  | 1 | 0.32 |
| Hap_57 | GCGAAATAACGTTCAAA | 1 | 1 |  |  |  |  |  |  |  |  |  | 2 | 0.64 |
| Hap_58 | GAGAAAAGTTGTTCGAT |  | 1 |  |  |  |  |  |  |  |  |  | 1 | 0.32 |
| Hap_59 | GGGAAATAACGTTCAAA | 5 | 3 |  |  |  |  |  |  |  |  |  | 8 | 2.56 |
| Hap_60 | GGGAAAAATTGTTCAAT |  | 1 |  |  |  |  |  |  |  |  |  | 1 | 0.32 |
| Hap_61 | ACCAAAAGTTGTTCGAT |  | 1 |  |  |  |  |  |  |  |  |  | 1 | 0.32 |
| Hap_62 | GAGAAATGACGCAGAAA |  | 1 |  |  |  |  |  |  |  |  |  | 1 | 0.32 |
| Hap_63 | GCGAAAAGTTGTTCGAT |  | 1 |  |  |  |  |  |  |  |  |  | 1 | 0.32 |
| Hap_64 | GAGAAAAGTTGCACAAA |  | 4 |  |  |  |  |  |  |  |  |  | 4 | 1.28 |
| Hap_65 | AAGAAATAACGCAGGAT |  | 1 |  |  |  |  |  |  |  |  |  | 1 | 0.32 |
| Hap_66 | GCCACAAGTTGTTCGAT |  | 1 |  |  |  |  |  |  |  |  |  | 1 | 0.32 |
| Hap_67 | GGGAAATAACGTTCAAT |  | 1 |  |  |  |  |  |  |  |  |  | 1 | 0.32 |
| Hap_68 | GAGAAATAACGTTCGAT |  | 2 |  |  |  |  |  |  |  |  |  | 2 | 0.64 |
| Hap_69 | GCGAAATAACGTTCGGA |  | 1 |  |  |  |  |  |  |  |  |  | 1 | 0.32 |
| Hap_70 | GACAAATAACGCACAAA |  | 1 |  |  |  |  |  |  |  |  |  | 1 | 0.32 |
| Hap_71 | ACGAAAAGTTGTTCGAT |  | 3 | 1 |  |  |  |  |  |  |  |  | 4 | 1.28 |
| Hap_72 | GCCACAAGTTGCTCGAT |  | 1 |  |  |  |  |  |  |  |  |  | 1 | 0.32 |
| Hap_73 | GGGAAATAACATTCAAA | 1 | 2 |  |  |  |  |  |  |  |  |  | 3 | 0.96 |
| Hap_74 | GGGGAATAACATTCGAT |  | 1 |  |  |  |  |  |  |  |  |  | 1 | 0.32 |
| Hap_75 | AAGAAATAACGTACAAA |  | 1 |  |  |  |  |  |  |  |  |  | 1 | 0.32 |
| Hap_76 | GCGGAATAATATTCAAT |  | 1 |  |  |  |  |  |  |  |  |  | 1 | 0.32 |
| Hap_77 | ACCACAAGTTGCTCGAT |  | 1 | 1 |  |  |  |  |  |  |  |  | 2 | 0.64 |
| Hap_78 | AAGAAAAGTTGCTCAAA |  | 2 | 1 |  |  |  |  |  |  |  |  | 3 | 0.96 |
| Hap_79 | ACCACAAGTTGTTCGAT |  | 2 | 2 |  |  |  |  |  |  |  |  | 4 | 1.28 |
| Hap_80 | GCCGAATGTTGCTCAAA |  | 2 |  |  |  |  |  |  |  |  |  | 2 | 0.64 |
| Hap_81 | AAGAAAAATCGTTCGAT |  | 1 |  |  |  |  |  |  |  |  |  | 1 | 0.32 |
| Hap_82 | GCCGAATAACACTCAAA |  | 1 |  |  |  |  |  |  |  |  |  | 1 | 0.32 |
| Hap_83 | GCCAAAAGTTGCTCAAA |  | 1 |  |  |  |  |  |  |  |  |  | 1 | 0.32 |
| Hap_84 | GCCACAAGTTGTTCGAA |  | 1 |  |  |  |  |  |  |  |  |  | 1 | 0.32 |
| Hap_85 | GGGAAAAGTTGCTCAAA |  | 1 |  |  |  |  |  |  |  |  |  | 1 | 0.32 |
| Hap_86 | GCCACATAACATTCGAT |  | 1 |  |  |  |  |  |  |  |  |  | 1 | 0.32 |
| Hap_87 | GAGGACTAACATTCGAT |  | 1 |  |  |  |  |  |  |  |  |  | 1 | 0.32 |
| Hap_88 | GAGAACTAACGTACGGT |  | 1 | 1 |  |  |  |  |  |  |  |  | 2 | 0.64 |
| Hap_89 | AAGAAAAGTTGTACGAT |  | 1 |  |  |  |  |  |  |  |  |  | 1 | 0.32 |
| Hap_90 | GCGAAATAACATTCGGT |  | 1 |  |  |  |  |  |  |  |  |  | 1 | 0.32 |
| Hap_91 | ACCAAAAGTTGCTCAAA |  | 3 | 1 |  |  |  |  |  |  |  |  | 4 | 1.28 |
| Hap_92 | GAGAACTAACGTACGGA |  | 1 |  |  |  |  |  |  |  |  |  | 1 | 0.32 |
| Hap_93 | GAGAACAGTTGCACAAT |  | 1 |  |  |  |  |  |  |  |  |  | 1 | 0.32 |
| Hap_94 | GCCAAATAACGCACAAA |  | 1 |  |  |  |  |  |  |  |  |  | 1 | 0.32 |
| Hap_95 | GCCAAAAAACGCAGGAT |  | 2 |  |  |  |  |  |  |  |  |  | 2 | 0.64 |
| Hap_96 | GCGAAAAGTTGTAGGAT |  |  |  |  |  | 1 |  |  |  |  |  | 1 | 0.32 |
| Hap_97 | GAGAAAAAACGCAGAAA |  |  |  |  |  | 1 |  |  |  |  |  | 1 | 0.32 |
| Hap_98 | ACGAAAAGTTGCTCGAT | 4 |  | 1 |  |  |  |  |  |  |  |  | 5 | 1.60 |
| Hap_99 | GCGAAATAACATTCAAA | 1 |  |  |  |  |  |  |  |  |  |  | 1 | 0.32 |
| Hap_100 | GCGGAATAACGTTCAAA | 1 |  |  |  |  |  |  |  |  |  |  | 1 | 0.32 |
| Hap_101 | GCCGAATAACGCACAAT | 2 |  |  |  |  |  |  |  |  |  |  | 2 | 0.64 |
| Hap_102 | GACAAATGACGCACAAT |  |  | 4 |  |  |  |  |  |  |  |  | 4 | 1.28 |
| Hap_103 | ACCGAATAACATTCAAA |  |  | 1 |  |  |  |  |  |  |  |  | 1 | 0.32 |
| Hap_104 | ACGAAAAAACGCTCAAT |  |  | 1 |  |  |  |  |  |  |  |  | 1 | 0.32 |
| Hap_105 | GCGAACAGTTGTACAAA |  |  | 1 |  |  |  |  |  |  |  |  | 1 | 0.32 |
| Hap_106 | ACCACAAGTTGCTCAAA |  |  | 1 |  |  |  |  |  |  |  |  | 1 | 0.32 |
| Hap_107 | ACCAAAAGACGTTCAAT |  |  | 1 |  |  |  |  |  |  |  |  | 1 | 0.32 |
| Hap_108 | GAGAAAAAACGCAGAAT |  |  | 3 |  |  |  |  |  |  |  |  | 3 | 0.96 |
| Hap_109 | GCGAACAGTTGCACAAA |  |  | 1 |  |  |  |  |  |  |  |  | 1 | 0.32 |
| Hap_110 | GACAAATGTTGCACGAT |  |  | 1 |  |  |  |  |  |  |  |  | 1 | 0.32 |
| Hap_111 | GCCAAAAAACGCAGAAT |  |  | 1 |  |  |  |  |  |  |  |  | 1 | 0.32 |
| Hap_112 | GACAAAAGTTGCTCGAT |  |  | 1 |  |  |  |  |  |  |  |  | 1 | 0.32 |
| Hap_113 | GCCGAATAACGTACGAT |  |  | 1 |  |  |  |  |  |  |  |  | 1 | 0.32 |
| Hap_114 | ACCACAAGATGCTCAAA |  |  | 1 |  |  |  |  |  |  |  |  | 1 | 0.32 |
| Total | | 63 | 92 | 35 | 11 | 4 | 3 | 2 | 20 | 18 | 36 | 28 | 312 | 100 |

Cam: Cambodia, Vie: Vietnam, Mal: Malaysia, Indo: Indonesia, Laos: Laos, PNG: Papua New Guinea, Bra: Brazil, Col: Colombia, Eth: Ethiopia, CM: China Myanmar, Tha: Thailand.
